# Phylogenomic analysis of novel Diaforarchaea is consistent with sulfite but not sulfate reduction in volcanic environments on early Earth

**DOI:** 10.1038/s41396-020-0611-9

**Published:** 2020-02-17

**Authors:** Daniel R. Colman, Melody R. Lindsay, Maximiliano J. Amenabar, Maria C. Fernandes-Martins, Eric R. Roden, Eric S. Boyd

**Affiliations:** 1 Department of Microbiology & Immunology, Montana State University, Bozeman, MT 59717 USA; 2 Department of Geoscience, University of Wisconsin, Madison, WI USA; 3 NASA Astrobiology Institute, Mountain View, CA USA

**Keywords:** Archaeal physiology, Biogeochemistry, Phylogenetics, Biogeochemistry, Metagenomics

## Abstract

The origin(s) of dissimilatory sulfate and/or (bi)sulfite reducing organisms (SRO) remains enigmatic despite their importance in global carbon and sulfur cycling since at least 3.4 Ga. Here, we describe novel, deep-branching archaeal SRO populations distantly related to other Diaforarchaea from two moderately acidic thermal springs. Dissimilatory (bi)sulfite reductase homologs, DsrABC, encoded in metagenome assembled genomes (MAGs) from spring sediments comprise one of the earliest evolving Dsr lineages. DsrA homologs were expressed in situ under moderately acidic conditions. MAGs lacked genes encoding proteins that activate sulfate prior to (bi)sulfite reduction. This is consistent with sulfide production in enrichment cultures provided sulfite but not sulfate. We suggest input of volcanic sulfur dioxide to anoxic spring-water yields (bi)sulfite and moderately acidic conditions that favor its stability and bioavailability. The presence of similar volcanic springs at the time SRO are thought to have originated (>3.4 Ga) may have supplied (bi)sulfite that supported ancestral SRO. These observations coincide with the lack of inferred SO_4_^2−^ reduction capacity in nearly all organisms with early-branching DsrAB and which are near universally found in hydrothermal environments.

## Introduction

Biological sulfate reduction accounts for the mineralization of 12–29% of the organic carbon that is delivered to the sea floor and thus has a large effect on the global sulfur and carbon cycles today and in the geologic past [1]. The dissimilatory reduction of sulfate (SO_4_^2−^) and (bi)sulfite (SO_3_^2−^) by sulfate/(bi)sulfite reducing organisms (SRO) is regarded as one of the most primitive of extant microbial metabolisms on the basis of isotopic data in the rock record [2] and their inferred physiological antiquity [3]. Specifically, active SRO have been documented in the early Archean via sulfur isotope analyses of sulfides in barite deposits from the Dresser Formation, North Pole, Australia dated to 3.47 Ga [2]. While initial evidence suggested these deposits to be sedimentary, more recent interpretations suggest that they are located in an ancient caldera and were formed by circulating hydrothermal fluids in sulfidic acid-sulfate type continental spring settings [4–7]. Thus, these observations suggest that SRO inhabited hydrothermal environments early in Earth history. Consequently, they also raise the question as to whether hydrothermal environments could have promoted the origin of SRO, and if so, what was the source of oxidized sulfur that supported the energy metabolism of these primitive taxa?

The most common form of oxidized sulfur in modern oxic environments is SO_4_^2−^ [8]. However, SO_4_^2−^ concentrations are estimated to have been far lower in marine systems on early Earth (<200 µM; [9, 10]) and only appear to have begun to accumulate to an appreciable extent near the end of the Archean. The increase in SO_4_^2−^ was concomitant with the rise of O_2_ ~2.4 Ga, although smaller O_2_-dependent increases may have also been present in localized areas [8, 9]. Potential abiogenic sources of SO_4_^2−^ prior to the rise of O_2_ include sulfur dioxide (SO_2_) released to the atmosphere by volcanic activity that was likely more widespread than it is today [11]. Photolysis of atmospheric SO_2_ to sulfuric acid (H_2_SO_4_) could have contributed SO_4_^2−^ to marine and terrestrial environments via rain-out of H_2_SO_4_ [12, 13] and the resultant SO_4_^2−^ may have been further concentrated by evaporation [14]. Alternatively, volcanic SO_2_ can ionize in water to form (bi)sulfite, (H)SO_3_^2−^. At temperatures greater than ~150 °C and in the presence of water, SO_2_ [(H)SO_3_^2−^ as solubilized] can disproportionate to form elemental sulfur (S^0^) or sulfide (H_2_S) and SO_4_^2−^ [15], a process that may have also allowed for localized enrichment of SO_4_^2−^ in hydrothermal environments. However, at temperatures <150 °C, SO_3_^2−^ is stable in aqueous solutions in the absence of strong oxidants (e.g., ferric iron ions or O_2_; [16]). As such, modeling studies have suggested that concentrations of SO_3_^2−^ may have equaled or exceeded those of SO_4_^2−^ in waters during the early Archean [17, 18] and this may have been especially true for anoxic, moderate temperature hydrothermal systems.

Several intriguing proposals have been put forth to reconcile the origin of SRO, the substrates that supported SRO energy metabolism, and environmental conditions likely to have been present at the time SRO emerged (>3.4 Ga). These include (i) an origin for SRO that were adapted to low SO_4_^2−^ levels [19], (ii) an origin for SRO only after sulfide oxidizing organisms (e.g., anoxygenic phototrophs) emerged since they can produce SO_4_^2−^ [20], and (iii) an origin for SRO as a SO_3_^2−^ reducer [21–23]. In potential disagreement with the first hypothesis is data indicating that fractionation of sulfur during SO_4_^2−^ reduction is minimal at the low SO_4_^2−^ concentrations that characterized early Earth environment (<200 µM or even much lower; [9, 19]). This raises questions as to how ^34^S depletion of sulfides in 3.4 Ga barites (up to ~21.1‰ relative to co-occurring sulfate [2]) would have occurred unless reduction took place in a localized setting that was enriched in SO_4_^2−^, such as a hydrothermal environment. The probability of the second hypothesis is discounted by recent phylogenetic analyses that suggest the capacity to fully oxidize H_2_S to SO_4_^2−^ via dissimilatory sulfite reductase (Dsr) in anoxygenic phototrophs evolved after the ability to reduce SO_4_^2−^ via Dsr [20]. The third hypothesis is appealing considering that energy has to be expended in the form of ATP to activate SO_4_^2−^ before it can be reduced to H_2_S while reduction of SO_3_^2−^ to H_2_S yields energy [24]. Nonetheless, evidence in support of this hypothesis in the form of extant, early-diverging SO_3_^2−^ reducing organisms do not yet exist.

Here, we describe novel archaeal populations distantly related to other Diaforarchaea (previously Thermoplasmata) from metagenome assembled genomes (MAGs) recovered from two moderately acidic, moderate temperature sulfur springs in Yellowstone National Park (YNP), Wyoming, USA: MV2-Eury and SJ3-Eury. Genomic, transcriptomic, cultivation-dependent, and geochemical observations suggest that these populations with deeply diverging Dsr proteins are supported by SO_3_^2−^ reduction (but not SO_4_^2−^ reduction). Results are discussed in relation to the insights that these newly identified SRO lineages reveal regarding the evolution of early SRO.

## Materials and methods

### Sample collection

Sediment samples for metagenomic analyses and water for geochemical analyses were collected from MV2 spring (44°36′36.14″, −110°26′21.95″) within the Greater Obsidian Pool Area (GOPA) of the Mud Volcano Geyser Basin of YNP, Wyoming in July 2014 (Supplementary Fig. S1). Details of sediment sampling, DNA extraction, 16S rRNA gene amplification, and geochemical measurements are described elsewhere [25]. To assess the persistence of the MV2-Eury population over time, MV2 sediments were again sampled in July 2016, November 2016, July 2017, June 2018, twice in July 2018, and once in October 2018 (Supplementary Table S1) using field methods described previously [25]. Additional geochemical data were gathered for MV2 (also referred to as “Figure 8” pool) from previously published studies [26, 27]. SJ3 spring is located within the Smokejumper Geyser Basin (N 44°24′57.42″, W −110°57′20.76″), and the sampling details, field measurements, and geochemistry of SJ3 are reported in detail elsewhere [28, 29].

### Metagenomic analyses

Total genomic DNA from MV2 sediments collected in July 2014 was shotgun sequenced at the Genomics Core Facility at the University of Wisconsin-Madison using the paired-end (2 × 250 bp) Illumina MiSeq platform. DNA fragments were prepared according to the manufacturer’s protocol. Reads were quality trimmed from both ends and Illumina adapters were removed using Trimmomatic v.0.35 and assembled with the metaSPAdes algorithm of SPAdes v.3.7.1, as previously described [28]. The qualities of the assemblies were assessed using various metrics. Contigs (>2.5 kbp) from the highest quality assembly (*K*-mer size = 127) were binned by tetranucleotide word frequency distribution patterns and a window size of 5 kbp using Emergent Self Organizing Map (ESOM) analyses [30]. MAGs within the ESOM were manually delineated using ESOM visualization tools from Databionic.

MAGs were then assessed for quality, contamination, and completeness using CheckM v.1.0.5 and refined as described previously [28]. Gene prediction and annotations were performed using Prokka v.1.11 [31]. Annotations of proteins that were of specific interest were further scrutinized using homology searches against NCBI’s nonredundant (nr) database, conserved domain database searches to identify protein family/domain conservation, identification of conserved amino acid motifs for certain protein-coding genes (PCGs), and phylogenetic analyses to determine homology to characterized proteins. The complete details of metagenomic sequencing of the SJ3 sediment community on the Illumina Hiseq 2500 Rapid platform, quality filtering, assembly, and genomic binning are provided elsewhere [28]. The assembled MV2-Eury contigs were used to recruit contigs >1 kbp from the SJ3 assembly exhibiting high nucleotide identity (≥90%). To confirm the genomic characteristics of MV2-Eury, a second metagenome was generated from MV2 sediments collected in October 2018 to reconstruct a second MV2-Eury MAG. The metagenome was sequenced on the Illumina NovaSeq platform with 2 × 150 bp paired-end reads, assembled, and binned using methods described elsewhere [32].

### Phylogenetic analyses

The genomic relatedness of the MV2- and SJ3-Eury MAGs was evaluated against other selected members of the major groups of Diaforarchaea using mean pairwise amino acid identity (AAI) calculations, as implemented in the CompareM software program (https://github.com/dparks1134/CompareM; v.0.0.23). Genomes associated with the Thermoplasmata class were retrieved from the Integrated Microbial Genomes and Microbiomes (IMG M) database [33], in addition to others associated with the major groups of Diaforarchaea identified in a recent analysis [34]. Only those genomes publicly released >2 years prior to retrieval, or otherwise associated with a published study, were used for the analyses. In addition, only genomes exhibiting >50% estimated completion were used for comparisons, as determined with CheckM. The pairwise AAI values were used to construct a heatmap in addition to a dendrogram of pairwise similarities that was constructed in the base R package (v.3.4.1) using the average clustering algorithm. Phylogenomic analyses were conducted by surveying euryarchaeal MAGs from MV2 and SJ3 along with representatives from previously described Diaforarchaea groups for single-copy housekeeping marker genes as previously described [28]. Complete details of phylogenomic analyses, including substitution model choice are provided in the Supplementary Text. In addition, genes encoding proteins of interest (DsrAB, AprA) were also subjected to phylogenetic reconstruction as described in detail in the Supplementary Text.

Phylogenetic analysis of 16S rRNA genes that represented the MV2-Eury and SJ3-Eury MAGs were conducted on aligned subject sequences and associated references using the Silva SEED (release 132) template alignment and the mothur software package v.1.39.5 [35] with the default aligner. All reference sequences under the “Thermoplasmata” class were retrieved from the Silva database [36] and screened such that only reference sequences >900 bp (*n* = 8270) were included in the alignments. In addition, near full-length reference 16S rRNA sequences were included from the NCBI genbank database based on close (>90%) nucleotide identity to those of the euryarchaeote MAGs. To reduce the size of the reference dataset, sequences were clustered into operational taxonomic units (OTUs) in mothur at the 97% nucleotide identity level and representative sequences were chosen for phylogenetic analyses (*n* = 1533). The aligned 16S rRNA gene dataset was then subjected to ML phylogenetic analyses using IQ-TREE [37] and the GTR + G + I nucleotide substitution model with 1000 bootstraps to evaluate node support.

Detection of MV2-Eury-like phylotypes across YNP hot spring communities was evaluated by BLAST searches of the 16S rRNA gene from the MV2-Eury MAG against previously published datasets [25, 26]. Positive detection was indicated by the identification of phylotypes with >97% nucleotide homology to the MV2-Eury 16S rRNA gene. All OTUs considered positive detections were summed for a given spring to represent the total relative abundance of MV2-Eury-like phylotypes in that spring. To evaluate estimated relative abundances with approaches less prone to population biases than PCR amplification, DsrA and RpoB homologs of MV2-Eury were searched against a published and in-house database of 33 metagenomic samples that we have generated from YNP springs (Supplementary Table S4). Positive detection of MV2-Eury sequences was defined as a match to either homolog at >95% AAI, and relative abundances were estimated based on MAG-binning and relative abundance estimates that were generated using methods described previously [28].

### Metabolic inference from reconstructed genomes

Protein-coding genes were surveyed from MAGs by referencing known pathways and homologs in closely-related, or model genomic assemblies. For the MV2- and SJ3-Eury MAGs for which there were no close taxonomic representatives, MV2-Eury pathways were assessed by surveying the PCGs for gene complements that corresponded to functionally characterized homologs in other Euryarchaeota or Crenarchaeota. BLASTp searches of protein homologs against the assemblies were used to identify additional pathways in the genomes (e.g., based on >30% amino acid homology and >60% coverage of query proteins), along with pathway annotation using the Kyoto Encyclopedia of Genes and Genomes database. Specifically, the presence or absence of DsrEFH homologs was evaluated by homology to those of *Chlorobaculum tepidum* (CT0855-CT0857), DsrD homologs of *Desulfovibrio vulgaris* subsp. *vulgaris* str. Hildenborough (DVU0404), Sat homologs of *Caldivirga maquilingensis* (Cmaq_0274), and AprAB homologs of *C. maquilingensis* (Cmaq_0272/0273).

To further validate the absence of Sat and AprA homologs that are necessary for dissimilatory SO_4_^2−^ reduction, Hidden Markov Model (HMM)-based searches were conducted using established HMM model packages in the GraftM program v.0.11.1 [38]. The default search settings were used with nonconservative identification threshold values to allow maximum identification of potential homologs. Given the small size of typical AprB (~90 aa) and its conserved biochemical features as a ferredoxin protein, an HMM-based model was not used to identify it within the assemblies due to the high likelihood of false positive hits. Further, HMMs based on Pfam database [39] models for additional marker genes involved in Dsr-based SO_4_^2−^ reduction (DsrD; PF08679.11) or S^0^ oxidation (DsrE/F and DsrH; PF02635.15 and PF04077.12, respectively) were used to confirm the absence of these proteins in the MAG assemblies with the Hmmer v3.1 program and default search settings (http://hmmer.org). To further evaluate whether gene fragments of Sat or AprA were present in the nucleotide assembly of the YNP-TEG MAGs, tBLASTn searches were used to query them using the NCBI tBLASTn tool. tBLASTn query homologs were used from *Vulcanisaeta, Caldivirga*, and *Pyrobaculum* strains that harbor the most closely-related DsrAB homologs to the MV2-/SJ3-Eury MAG DNA sequences.

### MV2 enrichment cultures

Sediments and spring water were sampled aseptically from MV2 spring in May 2018 and maintained under anaerobic conditions during the transit to the laboratory using previously described protocols [40]. The base salts medium contained NH_4_Cl (0.33 g l^−1^), KCl (0.33 g l^−1^), CaCl_2_⋅2H_2_O (0.33 g l^−1^), MgCl_2_⋅6H_2_O (0.33 g l^−1^), and KH_2_PO_4_ (0.33 g l^−1^) supplemented with peptone to achieve a final concentration of 0.03% (wt./vol.). The pH of the base salts medium was adjusted to 3.80 with concentrated hydrochloric acid. Medium (27.5 mL) was dispensed into 160 mL serum bottles and sterilized by autoclaving. Following autoclave sterilization and while still hot (~90 °C), 27.5 mL of filter sterilized spring water (pH 3.80), filter sterilized Wolfe’s vitamins (1 mL L^−1^ final concentration), and filter sterilized SL-10 trace metals (1 mL L^−1^ final concentration) were added. The bottles and their contents were deoxygenated by purging with sterile nitrogen gas (N_2_) passed over heated (210 °C) and hydrogen-reduced copper shavings. The serum bottles were sealed with butyl rubber stoppers and heated to 80 °C prior to the replacement of the headspace with 80%:20% H_2_:CO_2_. Just before inoculation, the medium was amended with anaerobic filter sterilized Na_2_SO_3_ solution (amended to a final concentration of 1 mM), equivalent concentrations of Na_2_SO_4_ solution, or without a terminal electron acceptor amendment as a control. The serum bottles were then inoculated with 1 mL spring-sediment/ water slurry sampled from MV2 spring and were incubated at 60 °C. Enrichment progress was monitored in terms of total sulfide production (S^2−^; proxy for SO_4_^2−^/SO_3_^2−^ reduction) using methods previously described [40]. Dissolved sulfide concentrations were determined with the methylene blue reduction method. Total sulfide production (dissolved and gaseous) was calculated using standard gas-phase equilibrium calculations as described previously [41].

### Expression of MV2 *dsrA* RNA

Expression of *dsrA* in the MV2-Eury population was investigated in six MV2 samples collected between July 2016 and July 2018 (Supplementary Table S1). A slightly modified version of the FastRNA Pro Soil-Direct kit (MP Biomedicals, Irvine, CA, USA) was used to extract RNA from MV2 sediments, as previously described [29]. Briefly, RNA extracts were DNAse-treated (Sigma-Aldrich, St. Louis, MO, USA) and subjected to PCR of 16S rRNA genes to determine if DNA persisted, as described previously [29]. cDNA was then synthesized using the iScript cDNA synthesis kit (Bio-Rad Laboratories, Hercules, CA, USA) in 20 µL reactions. Forward (5′ TGCCAGGGCATCACAAAAAG 3′) and reverse (5′ CCTTTCCTTTCTTGCAGCGTT 3′) primers were then designed to amplify a 394 bp fragment of the *dsrA* gene specific to the MV2-Eury MAG. Primer specificity was tested using genomic DNA from *Desulfovibrio vulgaris* and *D. gigas* as controls. Further, comparison of the primers against *dsrA* homologs from those two taxa and the most closely-related *dsrA* homologs from *Vulcanisaeta, Caldivirga*, and *Pyrobaculum* via alignments strongly suggests that they would not amplify homologs from any of the above taxa (Supplementary Fig. 2). Quantitative PCRs (qPCRs) of MV2-Eury *dsrA* transcripts were conducted with the SYBR Green Supermix (Bio-Rad Laboratories, Hercules, CA, USA) in a reaction volume of 20 µL and at an annealing temperature of 52 °C, as previously described [29]. To generate *dsrA* plasmid standards to relate template copy numbers to threshold amplification signals, PCRs with the above primers were conducted on template DNA. Amplicons were purified, ligated into pGEM plasmids (Promega, Madison, WI, USA), and cloned as previously described [29]. Transcript abundances were then normalized to gram dry weight sediment (gdws).

### Laboratory analysis of (bi)sulfite stability

(Bi)sulfite stability at various pH values and under anoxic conditions was determined using laboratory microcosm experiments. First, the pH of distilled H_2_O was adjusted to 2.0, 3.0, 4.0, 5.0, 6.0, and 7.0 with 0.5 M HCl or NaOH. The solutions were then boiled for 10 min and 9.5 mL aliquots were added to triplicate 24 mL serum bottles while under a running stream of N_2_ gas. Vials were capped, autoclaved, and purged with N_2_ for 20 min after autoclaving. The vials were transferred to a Coy anaerobic chamber (Coy, Grass Lake, MI, USA) where 0.5 mL of an anoxic solution of Na_2_SO_3_ (1 mM) was added to achieve a final concentration of 50 µM. The influence of pH on SO_3_^2−^ stability was assessed at 40 °C. The effect of temperature on SO_3_^2−^ stability was assessed in anoxic solutions with a pH of 4.0. The effect of oxygen on SO_3_^2−^ stability was also determined in solutions with pH 4.0 and at specified temperatures. However, the vials were not initially boiled under a stream of N_2_ gas, were allowed to cool to ambient temperature after autoclaving but prior to capping, and were then purged with a 20/80% mixture of O_2_/N_2_. In each assay, aliquots for SO_3_^2−^ were taken within an anaerobic chamber at 0 and 24 h post addition of Na_2_SO_3_ (with the exception of the oxic treatments). Sulfite concentrations were measured using a previously described colorimetric assay (abs. 570 nm) based on the reduction of fuchsin [42].

## Results and discussion

### Geochemical context of hot springs

MV2 (also referred to as “Figure 8”; 44°36′36.14″, −110°26′21.95″) is an acidic spring in the Greater Obsidian Pool Area of the Mud Volcano geyser basin of eastern-central YNP (Supplementary Fig. S1). At the time of initial sampling for metagenomics analysis in 2014, the temperature of MV2 was 62.0 °C and the pH was 3.8. Over the next 4 years of repeated sampling, the geochemistry of MV2 fluctuated (Supplementary Table S1). For example, the pH of MV2 waters ranged from 3.0 to 3.8 and the temperature ranged from 59 to 69 °C (Supplementary Table S1). Published data suggests even wider geochemical variance, with pH ranging as low as 3.0 and as high as 4.8 [25–27]. The relatively high levels of SO_4_^2−^ and Cl^−^ within MV2 waters are indicative of an acid-sulfate chloride spring (Supplementary Fig. S3), sourced by a mixture of a deep hydrothermal water end member enriched in Cl^−^ and a vapor-phase fluid end member enriched in SO_4_^2−^ via oxidation of sulfide (Supplementary Fig. S3) [43].

SJ3 spring is in the Smokejumper geyser basin within southwestern YNP (44°24′57.42″, −110°57′20.76″, Supplementary Fig. S1) [28]. At the time of sampling for metagenomics analysis in 2014, SJ3 waters exhibited a moderately acidic pH of 5.4 and a temperature of 61.9 °C. The geochemical composition of SJ3 (moderate SO_4_^2−^ levels and extremely low Cl^−^) is consistent with mixing of a vapor-phase fluid end member with surface-derived meteoric waters [29, 43] (Supplementary Table S1, Supplementary Fig. S3).

### Recovery of euryarchaeal MAGs from YNP hot spring metagenomes

Metagenomic characterization of the MV2 and SJ3 sediment communities from 2014 yielded five (Supplementary Text; Supplementary Table S2; Supplementary Fig. S4) and 82 [28] moderate to high-quality MAGs, respectively. A high-quality MAG estimated to be nearly complete (98% estimated completeness, Supplementary Table S3) was recovered from the MV2 community metagenome and contained a near full-length 16S rRNA gene (length = 1425 nt) that was 100% identical to those from the “Thermoplasmatales A10” group (Euryarchaeota phylum) that have been previously identified in MV2 sediments [25, 26] (Supplementary Figs. S5 and S6). This MAG, hereafter referred to as MV2-Eury, comprised 1.30 Mbp and 1384 predicted PCGs. An additional MAG recovered from the SJ3 hot spring community metagenome (hereafter SJ3-Eury) was represented by an unbinned 16S rRNA gene (length = 994 nt) that was 100% identical to that of MV2-Eury (Supplementary Figs. S5 and S6). Further, pairwise average amino acid and nucleotide identities (AAI/ANI, respectively) were nearly identical among the two Euryarchaeal MAGs (two-way AAI: 99.36% ± 1.50%, *n* = 1201 encoded proteins; two-way ANI: 99.08% ± 0.78%, *n* = 5599 alignments), suggesting a very high degree of genomic similarity, although unique subsets of proteins were present in each MAG. In addition, the two euryarchaeal MAGs also exhibited highly similar G + C contents (see Supplementary Table S3 for additional MAG statistics). The SJ3-Eury MAG was also estimated to be nearly complete (98% estimated completeness) but was smaller than that of MV2, comprising a length of 1.19 Mbp and 1254 predicted PCGs (Supplementary Table S3). The MAGs were estimated to have <1.0% redundancy (i.e., contamination) in single-copy marker genes (Supplementary Table S2), thus meeting the criteria of high-quality MAG drafts [44]. Lastly, a second MV2-Eury MAG was generated from sediments collected in 2018 that was effectively identical to the MV2-Eury MAG (two-way ANI: 99.85% ± 0.28%; unbinned 16S rRNA gene 100% identical to the 2014 MV2-Eury MAG), and is thus not discussed further.

**Fig. 1:**
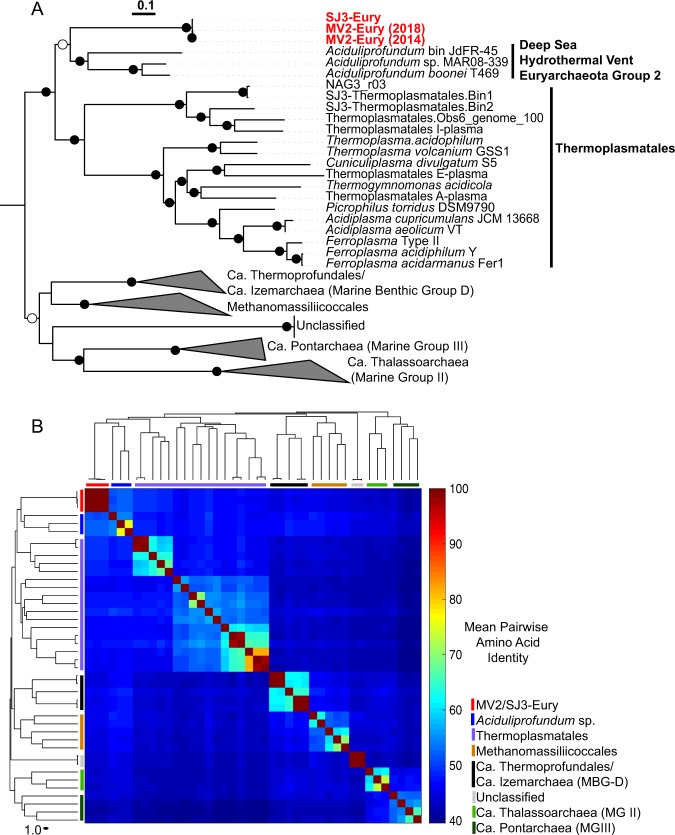
Phylogenomic comparisons of the novel euryarchaeal MAGs to other Diaforarchaea. **a** Maximum-likelihood (ML) phylogenomic reconstruction of the MV2/SJ3-Eury MAGs and other Diaforarchaea. The analysis comprised 103 housekeeping protein homologs conserved across Archaea. Branch length is relative to the scale provided at the top indicating the expected number of substitutions per site. Bootstrap values >90 for nodes are indicated by black circles while those between 50 and 90 are denoted by white circles. Values are not depicted for nodes with bootstrap support <50 (out of 100 ML bootstraps). *Archaeoglobus fulgidus* and *Methanobacterium formicicum* were used as the outgroups (not shown). Clade-level triangles indicate the phylogenetic diversity within each group via side lengths that are proportional to the distances between the clade’s closest and furthest taxa. **b** Heatmap showing the pairwise average amino acid identities (AAI) between members of the major groups of Diaforarchaea that were used in the phylogenomic analysis in **a**. Groups are indicated by colored lines based on the legend at the bottom right. Pairwise AAI values are colored according to the scale on the right. The dendrogram was constructed based on hierarchical clustering of the pairwise average AAI values and the branch length is proportional to the scale at the bottom of the dendrogram.

Phylogenomic analyses distinguished the MV2/SJ3 MAGs from other higher-order clades within the recently proposed euryarchaeal superclass Diaforarchaea [45] (previously referred to as Thermoplasmata; Fig. 1a, b) and better resolved the branching of nodes within the Diaforarchaea compared to the 16S rRNA gene analyses (Supplementary Figs. S5 and S6). Pairwise comparisons among other members of the major Diaforarchaea groups indicated that the MV2-/SJ3-Eury MAGs shared <60% mean AAI with all members of the Diaforarchaea and <50% AAI with members of the most closely-related phylogenetic clade (Fig. 1b), the *Aciduliprofundum* genus (previously known as the Deep Sea Hydrothermal Vent Euryarchaeota group 2 [DHVE2] Archaea). Such values are consistent with a class or order-level distinction from the *Aciduliprofundum* based on previously published suggestions [46]. Consequently, the analyses indicated that the MV2- and SJ3-Eury MAGs represented members of a previously uncharacterized group of Diaforarchaea that are distantly related to the *Aciduliprofundum* spp., which together comprise an outgroup to acidophilic Thermoplasmatales-related Archaea (Fig. 1a, b). The moderately acidophilic, S^0^/ferric iron reducing DHVE2 Archaea were first isolated from deep sea hydrothermal vents [47], and DHVE2 cultivars or MAGs are thus far only known from such environments. This biogeographic distinction suggests that the MV2/SJ3-Eury clade represents a terrestrial hydrothermal lineage that is ecologically distinct from currently characterized DHVE2. In support of this assertion, all publicly available 16S rRNA gene phylotypes associated with the MV2/SJ3-Eury phylotypes were recovered from terrestrial hydrothermal springs/pools (Supplementary Figs. S5 and S6), with no close representatives from marine vents. Specifically, 16S rRNA gene phylotypes closely related to this lineage have also been detected in acidic/moderately acidic and moderate temperature hot springs in geothermal fields in YNP, Japan, Kamchatka, Taiwan, and elsewhere (Supplementary Fig. S6), suggesting that the group is widely distributed among terrestrial hydrothermal systems with these characteristics. The clade is thus referred to hereafter as the YNP terrestrial euryarchaeotal group (YNP-TEG).

### A new taxonomic clade of SRO

**Fig. 2:**
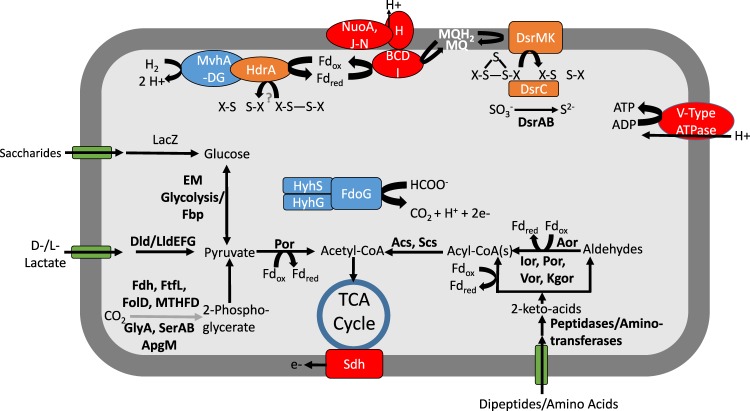
Reconstructed core metabolic model for the YNP-TEG MAGs. Question marks indicate unknown metabolic transformations, while gray lines indicate hypothesized metabolic pathways. Abbreviations are as follows: Dsr dissimilatory (bi)sulfite reductase, MQ menaquinol, Nuo archaeal-like ferredoxin-dependent Nuo complex, Hdr heterodisulfide reductase, Mvh F_420_ non-reducing [NiFe] hydrogenase (Group 3c type), LacZ beta-galactosidase, EM Embden–Meyerhoff glycolysis, Dld/Lld D/L-lactate dehydrogenase, Fdh formate dehydrogenase, FtfL formate-tetrahydrofolate ligase, FolD methylenetetrahydrofolate dehydrogenase, MTHFD methylenetetrahydrofolate dehydrogenase, GlyA glycine hydroxymethyltransferase, Ser D-3-phosphoglycerate dehydrogenase, Apg 2,3-bisphosphoglycerate-independent phosphoglycerate mutase, Fd ferredoxin, Sdh succinate dehydrogenase, Por pyruvate ferredoxin oxidorectase, Acs acetyl-CoA synthase, Scs succinyl-CoA synthetase, Aor aldehyde ferredoxin oxidoreductase, Ior indolepyruvate ferredoxin oxidoreductase, Vor 2-ketoisovalerate ferredoxin reductase, Kgor 2-ketoglutarate ferredoxin oxidoreductase, Fdo formate dehydrogenase, Hyh NADP(H)-coupled bidirectional [NiFe] hydrogenase (Group 3b type).

Reconstruction of metabolic pathways involved in energy conservation for the YNP-TEG group indicated that the organisms are putatively capable of SO_4_^2−^/SO_3_^2−^ reduction coupled to hydrogen and/or organic acid (e.g., lactate, acetate, and formate) oxidation (Fig. 2, Supplementary Text, Supplementary Dataset 1). Both MV2 and SJ3 MAGs contained adjacent *dsrA* and *dsrB* protein encoding genes that were distantly related to other DsrAB. Sequence alignment of DsrA confirmed the presence of characteristic siroheme binding (CX_5_CX_n_CX_3_C; Supplementary Fig. S7) and [Fe_4_S_4_] cluster binding (CX_2_CX_2_C; Supplementary Fig. S7) motifs [48]. DsrAB are catalytically active in both the forward (reductive) and reverse (oxidative) directions [49]. The former is used during SO_4_^2−^/SO_3_^2−^ reduction in the six bacterial and archaeal lineages with characterized SRO: the Archaeoglobales, Deltaproteobacteria, Firmicutes, Crenarchaeota, Nitrospirae, and Thermodesulfobacteria [50]. In contrast, the latter is used by sulfur oxidizing organisms primarily affiliated with anoxygenic phototrophs (i.e., Chlorobi) and the Proteobacteria [50] and may also be used for sulfur disproportionation in *D. alkaliphilus* [51].

Three primary phylogenetically distinct clusters of DsrAB have been previously identified, and these coherently group proteins with physiological biases for DsrAB directionality. These include the (1) bacterial (and Archaeoglobales) reductive DsrAB, (2) bacterial oxidative DsrAB, and (3) crenarchaeal reductive DsrAB (nomenclature following [49]). It should, however, be noted that a recent characterization of the elemental sulfur (S^0^)/polysulfide disproportionating *D. alkaliphilus* deltaproteobacterium revealed the capacity for S^0^/polysulfide oxidation or disproportionation that is inferred to be catalyzed by a bacterial reductive type DsrAB, suggesting that the directionality of DsrAB based on phylogenetic clustering may not always be coherent [51]. Nevertheless, the majority of characterized SRO exhibit phylogenetic placements of DsrAB homologs that are consistent with their directionality, and it is not yet clear if the above example is unique to *D. alkaliphilus* or S^0^/polysulfide disproportionators, or if it is more widespread. In addition, the recent suggestion of putative SRO or S^0^ oxidizing lineages from uncultured candidate division MAGs based on bioinformatics data could potentially further confound the inference of DsrAB catalysis from sequence-based homology [50]. Physiological characterization of these uncultured taxa and their capacity for SO_4_^2–^/SO_3_^2−^ reduction or S^0^ oxidation will be necessary to fully discern whether they validate the use of DsrAB phylogenies to support catalytic directionality.

**Fig. 3:**
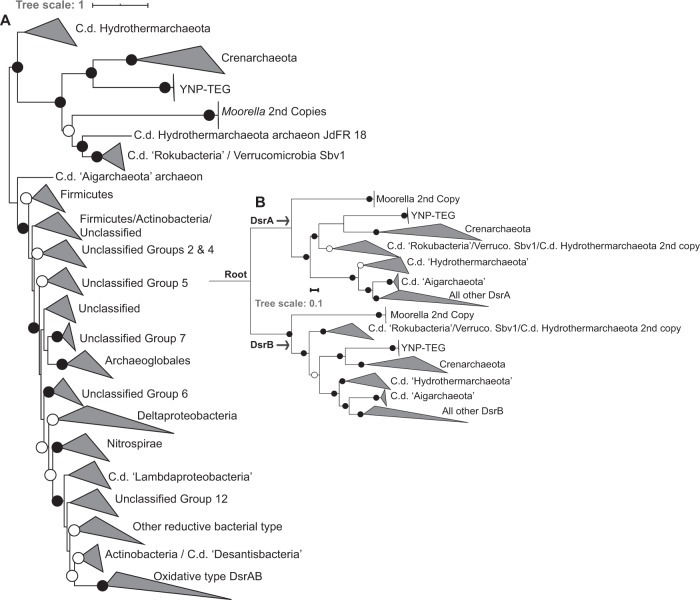
Maximum-Likelihood phylogenetic reconstruction of YNP-TEG DsrAB subunit homologs. **a** Unrooted phylogenetic reconstruction of a concatenated DsrAB alignment block (alignment length = 1230 positions, *n* = 1218 homolog pairs). The tree is displayed with an artificial midpoint-rooting visualization. Branches are identified according to major taxonomic/functional groups (following the classification scheme of [49]). Bootstrap values >90 for clade-level bifurcations are indicated by black circles while those between 50 and 90 are denoted by white circles. Values are not depicted for nodes with bootstrap support <50. Branch length is relative to the scale provided at the top indicating the expected number of substitutions per site. The clade-level triangles indicate the phylogenetic diversity within each group via side lengths that are proportional to the distances between the clade’s closest and furthest taxa. **b** Paralogous rooting of the DsrA (top) and DsrB (bottom) subunits using a subset of taxa (*n* = 62) from the major lineages shown in the more expansive phylogenetic analysis in **a**. Bootstrap values are depicted the same as in panel **a**. Branch length is relative to the scale provided at the left indicating the expected number of substitutions per site. The root is labeled where the DsrA/DsrB subunits are presumed to have originated by a gene duplication prior to expansion and radiation. The clade-level triangles indicate the phylogenetic diversity within each group via side lengths that are proportional to the distances between the clade’s closest and furthest taxa.

An unrooted phylogenetic analysis of concatenated YNP-TEG DsrAB homologs (*n* = 1218, comprising homologs from previously described datasets [49, 50]) indicated that they form an outgroup to reductive crenarchaeal DsrAB (Fig. 3a, Supplementary Fig. S8). Little is known of crenarchaeal DsrAB and crenarchaeal SRO, in general, with only four demonstrated SO_4_^2^/SO_3_^2−^ reducing genera in the phylum, all within the Thermoproteales order. SO_4_^2−^ reduction has been suggested or observed in the *Vulcanisaeta*, *Caldivirga*, and *Thermoproteus* [52–54], while SO_3_^2−^ reduction (but not SO_4_^2−^ reduction) has been observed in *Pyrobaculum* [55]. Further, SO_4_^2−^ reduction has been suggested in Thaumarchaeota MAGs recovered from YNP springs, wherein the thaumarchaeal DsrAB show very high homology to those from *Vulcanisaeta* [32]. Thus, phylogenetic relatedness of the DsrAB homologs among these strains and the YNP-TEG MAGs (albeit with amino acid identities <45%) suggests that the latter are involved in SO_4_^2−^ or SO_3_^2−^ reduction. The unrooted phylogenetic analyses also suggested an early DsrAB evolutionary trifurcation, with one branch comprising the YNP-TEG/crenarchaeal DsrAB homologs. The second branch comprised duplicate DsrAB copies within *Moorella* spp. genomes and uncultivated organisms within the candidate division (c.d.) “Rokubacteria”, c.d. “Hydrothermarchaeota”, and Verrucomicrobia (Fig. 3a; Supplementary Fig. S8, Supplementary Dataset 2), which have all previously been shown to branch deeply in DsrAB phylogenies [50]. The third branch comprised the canonical reductive and oxidative type DsrAB, in addition to deeply-branching archaeal homologs in the “Aigarchaeota” and “Hydrothermarchaeota” candidate divisions (c.d.).

DsrA and DsrB share significant homology and are likely to have arisen from gene duplication prior to the radiation of SRO [48, 49]. Thus, to better discern the phylogenetic placement among early-branching DsrAB, paralogous rooting was conducted for DsrA and DsrB (Fig. 3b). These analyses indicated that the YNP-TEG archaeal DsrAB lineage is among the earliest evolving DsrA and DsrB groups, which is consistent with the basal branching of crenarchaeal DsrA and DsrB in other analyses [49, 50]. Identical basal-branching topologies were recovered in phylogenetic reconstructions using multiple Maximum-Likelihood (ML) algorithms (RAxML and IQ-TREE) and/or with the use of site-heterogeneous amino acid frequency mixture models that have been shown to mitigate long branch attraction of rapidly evolving lineages [56]. Specifically, the placement of the branches shown in Fig. 3b were invariant to ML algorithm or substitution scheme. Given the deep-branching of the YNP-TEG DsrAB, monophyly with Crenarchaeota/Thaumarchaeota DsrAB, and the general concordance of Thermoproteales DsrAB with taxonomic phylogenies suggesting vertical transfer (Supplementary Fig. S9), one of several evolutionary scenarios could explain the presence of SRO in the Diaforarchaea. This includes (1) DsrAB were horizontally transferred into an ancestor of the YNP-TEG group from an organism harboring primitive DsrAB homologs, followed by a subsequent transfer to the ancestor of the Thermoproteales order comprising *Vulcanisaeta, Caldivirga*, *Thermoproteus*, and *Pyrobaculum* (and a later transfer to Thaumarchaeota; [32]), (2) DsrAB were present in the most recent common ancestor of the YNP-TEG and the Thermoproteales and were subsequently lost among other euryarchaeal and crenarchaeal lineages, or (3) DsrAB were acquired relatively recently via a horizontal transfer into the YNP-TEG lineage, and separately into the Thermoproteales. The second evolutionary scenario is highly implausible considering the breadth of phylogenetic distance separating the Diaforarchaea within the Euryarchaeota and the Thermoproteales within the Crenarchaeaota (Supplementary Fig. S9). In addition, if the third scenario was likely, the DsrAB homologs of the YNP-TEG and Thermoproteales lineages would be phylogenetically nested within other ancestral donor lineages, as is well-documented for DsrAB in the euryarchaeal order Archaeoglobales [49].

Consequently, the combined phylogenetic analyses suggest that the YNP-TEG group diverged from the S^0^/Fe(III) reducing DHVE2 group after the divergence of the combined YNP-TEG/DHVE2/Thermoplasmatales group from other Diaforarchaea. This taxonomic divergence was likely associated with either the maintenance of ancestral SO_3_^2−^ reduction PCGs in the YNP-TEG lineage or acquisition of these PCGs from an unidentified or extinct lineage prior to the early divergence of all DsrAB into the three contemporary higher-order groups described above. Nevertheless, identification of additional deep-branching archaeal DsrAB, if these indeed exist, would improve the resolution of these potential evolutionary scenarios.

### Core metabolism of the YNP-TEG group

The YNP-TEG MAGs encode a number of additional proteins commonly involved in the dissimilatory SO_3_^2−^ reduction pathway (Supplementary Dataset 1) including DsrC (with conserved C-terminal cysteine residues; Supplementary Fig. S7) that acts as a mediator of SO_3_^2−^ reduction via the formation of a trisulfide bond [57]. It has been proposed that the membrane bound DsrMK(JOP) complex couples the reduction of the trisulfide bond to menaquinol oxidation [57]. This reaction would result in the downstream transport of protons (e.g., via Nuo) across the plasma membrane that could then be used in ATP synthesis (Fig. 2).

Neither of the YNP-TEG assemblies encoded identifiable homologs of sulfate adenylyl transferase (Sat) or adenosine-5′-phosposulfate reductase AB subunits (AprAB) that catalyze the activation of SO_4_^2−^ to adenosine-5′-phosphosulfate (APS) and APS reduction to SO_3_^2−^, respectively. HMM-based searches of Sat proteins did not yield any positive matches, while those with AprA models yielded only potential orthologs that upon further inspection did not exhibit conserved biochemical features of characterized AprA of SRO (discussed further below). Moreover, the absence of these proteins was confirmed using tBLASTn searches of homologs from the most closely-related taxa with DsrAB homologs, *Vulcanisaeta distributa*, *Caldivirga maquilingensis*, and *Pyrobaculum islandicum*. While AprA-like proteins were detected in both YNP-TEG assemblies, as indicated above, phylogenetic analyses and the lack of conserved AprA-typical residues indicated that they did not share homology with AprA from SRO that genomically co-localize with SO_4_^2−^ reduction genes (*qmoABC* and/or *sat*), but rather belonged to a divergent family of paralogous proteins (Supplementary Fig. S10, Supplementary Text). The lack of identifiable Sat and AprAB homologs in MV2-Eury and SJ3-Eury MAGs, despite being nearly (98%) complete and from springs separated by >100 km and from different years suggests that these organisms lack the ability to activate SO_4_^2−^ and reduce APS. Moreover, a second MV2-Eury MAG produced from sediments collected from MV2 in November 2018 (estimated completeness: 98%; ~100% ANI to the 2014 MV2-Eury MAG) confirmed the lack of Sat and Apr homologs. This is unlikely to be an artifact of genome binning, since a survey of all unbinned contigs and other MAGs from the 2014 MV2 and SJ3 metagenome did not yield homologs that could be attributed to the two organisms (Supplementary Dataset 3) combined with three nearly identical, high-quality, effectively complete MAGs produced from different springs and from multiple years. Lastly, a screen of the MV2-Eury and SJ3-Eury MAGs indicated the absence of other previously published marker gene homologs for S^0^/sulfide oxidation (Sqr; Sox; Fcc; DsrEFH) and SO_4_^2−^ reduction (DsrD) (Supplementary Text).

Consistent with the conclusion that YNP-TEG are unlikely to be capable of SO_4_^2−^ reduction, enrichment cultures from MV2 spring sediments sampled in May 2018 that were supplied with HSO_3_^−^ (most abundant form at pH 4–5) as the oxidant and peptone as a source of carbon and reductant (in addition to H_2_) yielded considerable sulfide (final concentration of 629 µM) after incubation for several weeks at 60 °C. However, despite numerous attempts, successful transfer of initial enrichments have not been achieved, disallowing more complete characterization. In contrast, enrichment cultures provided with SO_4_^2−^ did not produce sulfide over this time interval, including those amended with H_2_/CO_2_, lactate, peptone, or H_2_/CO_2_ plus peptone or H_2_/CO_2_ plus lactate. Importantly, samples are acidified in the methylene blue assay used to detect sulfide, which allows for detection of metal sulfides that might have been generated by SRO in sediments included as inoculum. Thus, binding of sulfide in mineral matrices would not likely affect the sulfide production values observed here. These results are thus consistent with predictions from genomic data and suggest the capacity for HSO_3_^−^ (but not SO_4_^2−^) reduction in MV2 populations. We attribute the activity observed in the enrichments from MV2 to MV2-Eury populations, since the only other putative SRO identified in the MV2 sediment community was a Thermoproteales-associated organism with potential capacity for SO_4_^2−^ reduction via Sat and AprAB (Supplementary Table S3, Supplementary Dataset 3). Nevertheless, because the inoculation sample was not taken at the same time as the metagenomic analysis sample, it is possible that other low-abundance SO_4_^2−^ reducers could have been present when sampling for cultivation. However, given that the same sediment samples were used to inoculate both the SO_4_^2−^ and SO_3_^2−^ cultures, it is unlikely that an SRO would be active in SO_3_^2−^ reduction, but not SO_4_^2−^ reduction, unless it was an obligate SO_3_^2−^ reducer, as is inferred for the MV2-Eury populations. Subsequent ongoing mixed-population enrichments from samples collected in Fall 2019 have also indicated the presence of SO_3_^2−^ reduction activity after months of incubation. Moreover, the presence of the MV2-Eury populations in these cultures was confirmed based on PCR-based detection of MV2-Eury-specific *dsrA* using population-specific primers described in the materials and methods.

Possible reductants capable of coupling with SO_3_^2−^ reduction in the YNP-TEG organisms are hydrogen (H_2_) or organic acids (e.g., formate, acetate, and lactate; Fig. 2, Supplementary Text). In addition, the MAGs encoded numerous peptidases and aminotransferases, in addition to dipeptide and single amino acid transporters. This indicates a general ability to metabolize cellular and extracellular proteins and amino acids via heterotrophic metabolism (Fig. 2) and is potentially consistent with the aforementioned enrichment data. Further, a hypothesized CO_2_ fixation pathway was present in the YNP-TEG MAGs (Supplementary Text), but it is unclear if the pathway is active and whether these organisms can grow autotrophically or mixotrophically.

### Ecological distribution of the YNP-TEG

**Fig. 4:**
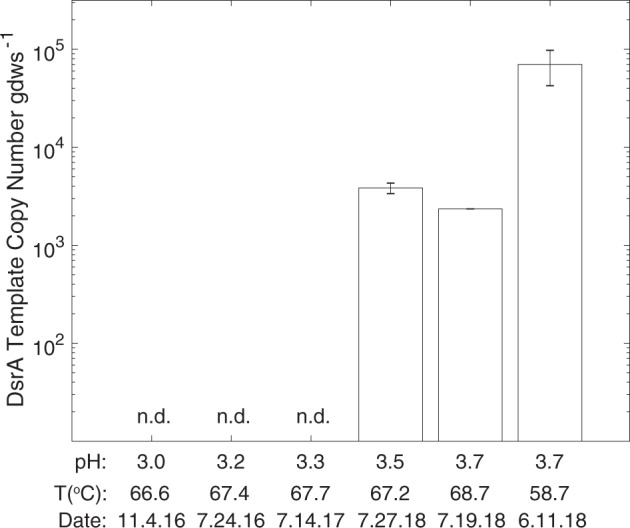
Abundance of MV2-Eury specific *dsrA* transcripts within MV2 sediments over multiple sampling intervals between 2016 and 2018. The mean values of triplicate qPCR assays are shown and error bars indicate standard errors. Sediment samples are arranged by MV2 spring pH with corresponding spring temperatures and sampling dates below each. n.d. indicates that no DsrA expression was detected in the sediment community RNA.

MV2-Eury is a consistent member of the sediment community of MV2, as evidenced by the detection of 16S rRNA genes with homology to this MAG in samples taken between 2010 and 2018 [25, 26] despite fluctuating geochemistry over these time intervals (Supplementary Table S1). Intriguingly, MV2-Eury populations were present at multiple sampling events between 2016 and 2018 over several seasons as shown by detection of DsrA homologs in DNA extracts (data not shown). However, active transcription of DsrA was only detected in sediments when MV2 exhibited a more moderately acidic pH (>3.5) in June–July 2018 than at any other sampling period over the previous two years (e.g., when spring pH < 3.5; Fig. 4). Given the high abundances of MV2-Eury populations in samples taken when the pH of MV2 was >3.5 [25, 26], in addition to the presence of a nearly identical MAG in SJ3 spring that exhibits a higher pH of 5.4, it is likely that the YNP-TEG generally occupy a higher pH niche that is variably present in the geochemically dynamic MV2 spring.

**Fig. 5:**
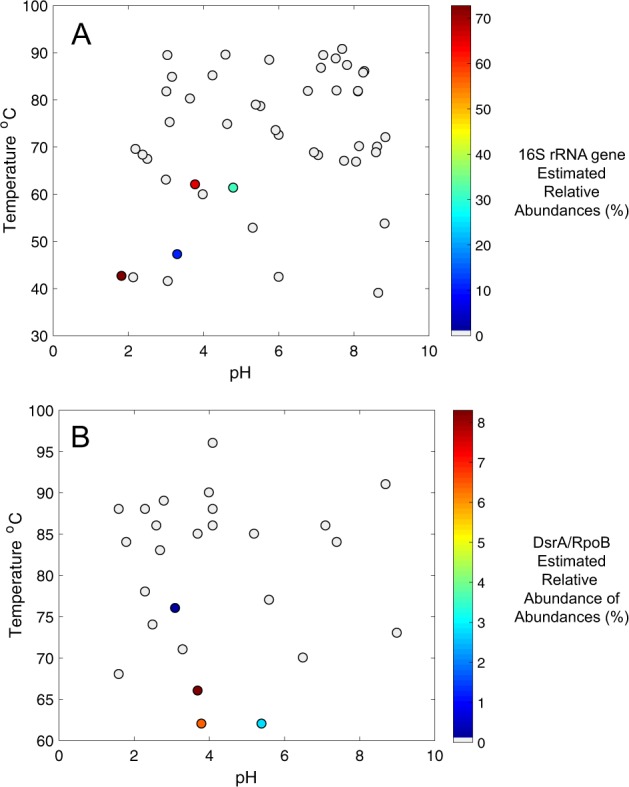
Distribution of YNP-TEG-like phylotypes among YNP hot spring communities. **a** 16S rRNA gene distribution revealed by comparison against 32 YNP hot spring communities described in Colman [26] and 15 communities evaluated in Colman et al. [25]. Positive detection was defined as when 16S rRNA gene identities >97% were identified relative to the MV2-Eury 16S rRNA gene. Points are colored by estimated relative abundance, as given by the scale on the right. Gray circles indicate the lack of detection in >0.01% relative abundance. **b** Inferred distribution of YNP-TEG-like phylotypes using an in-house database of 33 chemosynthetic hot spring community metagenomes that we have previously generated from YNP springs (Supplementary Table S4). Relative abundances were estimated based on DsrA/RpoB amino acid identities >95% to the MV2-Eury phylotype in MAG-binned contigs, followed by relative abundance estimates calculated by read-mapping as described in the materials and methods. The distribution of MV2-Eury-like DsrA and RpoB homologs were identical, and thus only one scatter plot is shown. Of the four metagenomes with positive YNP-TEG detection, two were MV2 from different years and another was SJ3 (Supplementary Table S4). Note that the samples in **a** and **b** derive from different datasets and do not necessarily represent the same springs. Likewise, the temperature and relative abundance scales differ between the two panels to emphasize within-dataset variation.

A survey of MV2-Eury-like 16S rRNA gene phylotypes (>97% nucleotide identity) among previously published archaeal community 16S rRNA gene datasets from 48 YNP springs [25, 26] revealed a patchy distribution in thermal springs with moderate temperatures and moderately acidic pH (Fig. 5a). Specifically, MV2-Eury phylotypes were only present in >1.0% relative abundance in five of 48 YNP springs with available data (two of which included MV2 from two different years). The pH of these spring waters ranged from 1.8 to 5.4 and the temperatures ranged from 42.5 to 62.0 °C. Likewise, a survey of MV2-Eury homologs (DsrA and RpoB) among 33 chemosynthetic metagenomes we generated from YNP springs as part of this and other projects ([28, 32, 58]; Supplementary Table S4), revealed a similar distribution (pH: 3.1–5.4; Temperature: 62–76 °C), albeit with lower estimated population abundances than the 16S rRNA gene BLAST searches (Fig. 5b). Relative abundances estimated from 16S rRNA gene abundances are known to be problematic for several reasons including overestimation due to multiple 16S rRNA gene copies within genomes [59], PCR primer bias [60], and the inability of certain PCR primers to amplify certain taxa, an issue that is particularly exacerbated for Archaea, and specifically Crenarchaea [61], that are often abundant in thermal springs. Thus, the estimated abundances of the MV2-Eury-like phylotypes in the metagenomic datasets are likely to be more accurate estimates of in situ abundances. Nevertheless, the pH and temperature ranges where YNP-TEG-like populations were identified were broadly consistent in both datasets, despite being derived from different thermal springs. As discussed below, the stability and chemical speciation of SO_3_^2−^ shifts considerably in the pH range of ~3–6, which may ultimately bound the niche space occupied by SO_3_^2−^ reducing YNP-TEG within hydrothermal systems.

### (Bi)sulfite prevalence in hydrothermal environments

SO_3_^2−^ is extremely unstable in modern oxic environments and rapidly oxidizes abiotically to SO_4_^2−^ [16, 62]. However, SO_3_^2−^ (primarily HSO_3_^−^ in aqueous solutions with pH 2.0–5.5; Supplementary Fig. S11; [63]) can be measured in appreciable concentrations in some hydrothermal springs [64]. At temperatures <150 °C [15], volcanic SO_2_ can hydrate and ionize to form SO_3_^2−^. Further, thiosulfate (S_2_O_3_^−^) produced during oxidation of sulfide [62] is unstable at pH < 4.0 and degrades to S^0^ and SO_3_^2−^ [62]. Thus, several abiotic mechanisms exist to supply SO_3_^2−^ in volcanically-influenced hydrothermal environments. Indeed, SJ3 and other springs within the Smokejumper geyser basin and MV2 within the Mud Volcano geyser basin exhibit some of the highest volcanically-sourced gas concentrations in YNP [29]. Thus, volcanic gas is especially abundant in these two areas of YNP, which may deliver a consistent supply of SO_3_^2−^ to these springs via SO_2_ or H_2_S processing.

**Fig. 6:**
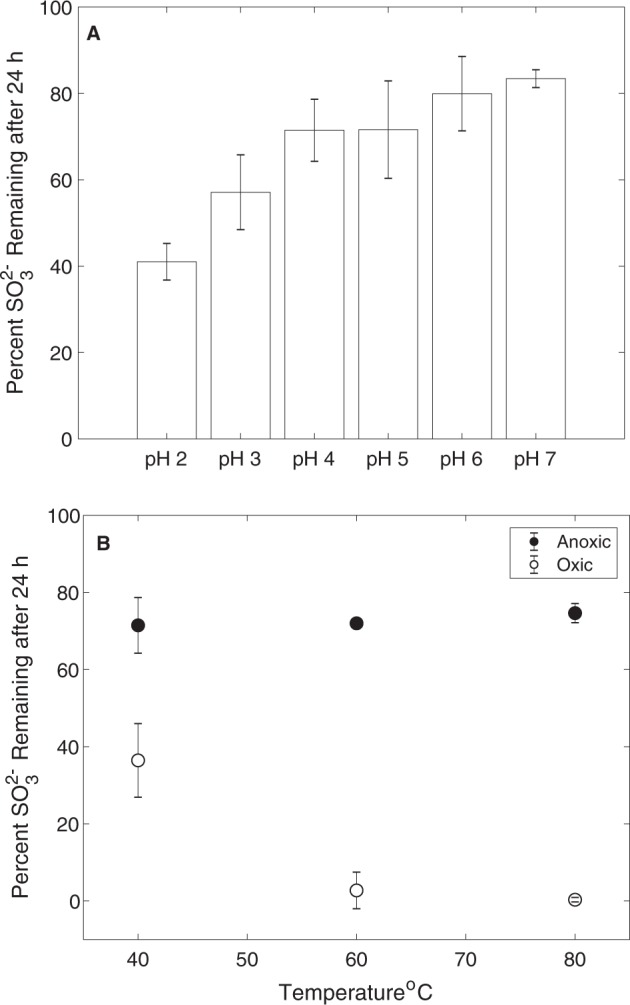
(Bi)sulfite stability under varying simulated geochemical conditions. **a** (Bi)sulfite stability under anoxic conditions and at pH values that encompass the range observed in MV2 and SJ3 springs. The mean percentage of SO_3_^2−^ remaining 24 h after adding Na_2_SO_3_ to a concentration of ~50 µM is shown for triplicate microcosms prepared at each pH interval and incubated at 40 °C, while error bars show the standard deviation of the assays. **b** The effect of temperature and oxygen (20% O_2_, 80% N_2_) exposure on the stability of (bi)sulfite at pH 4. Microcosms were prepared and measured as in Fig. 6a, but at varying temperatures and with or without preparation under anoxic conditions.

The primary mechanism leading to SO_3_^2−^ oxidation to SO_4_^2−^ in contemporary hydrothermal environments is via O_2_^−^ or Fe^3+^ ions as oxidants [16], although atmospheric photolysis of SO_2_ could potentially contribute SO_3_^2−^ to near surface waters [2]. Thus, in the absence of either O_2_ or Fe^3+^ ions, SO_3_^2−^ should be relatively stable, in particular in subsurface environments or within spring sediments where MV2-Eury is predominantly found [25]. Indeed, laboratory experiments conducted here indicate that SO_3_^2−^ solutions that were prepared anoxically maintained >80% of the original SO_3_^2−^ after 24 h incubations (Fig. 6a), while those incubated in the presence of O_2_ had very little SO_3_^2−^ remaining after incubation over the same period (Fig. 6b). SO_3_^2−^ stability was more pronounced in solutions with moderately acidic pH, with ~70–80% of SO_3_^2−^ remaining after anoxic incubations for 24 h at pH between 4 and 6. In addition, increased temperatures (i.e., ≥60 °C) resulted in increased SO_3_^2−^ oxidation under oxic conditions, but not under anoxic conditions. Thus, hot springs that harbor moderately acidic pH, are of moderate temperature, are anoxic or suboxic, and that feature high inputs of volcanic gas favor SO_3_^2−^ stability that, in turn, increases its bioavailability for organisms such as YNP-TEG. Importantly, these are distinguishing characteristics of the sulfur-rich MV2 and SJ3 springs, but such springs are relatively uncommon in the YNP geothermal system [32], which may explain the relatively limited ecological distribution of YNP-TEG organisms among YNP springs (Fig. 5a, b).

### Implications for the origins of SRO

The identification of deeply-branching DsrAB homologs in organisms that characteristically inhabit moderately acidic hot springs prompts the question as to whether such environments might have existed at the time that SRO are thought to have originated. SRO are thought to have been present as early as 3.47 Ga, based on sulfur isotopic data from sulfides in barite deposits from the Dresser Formation [2]. Recent data suggest that these deposits formed in an ancient volcanic caldera and several of these, in particular the barites, were likely formed by circulating hydrothermal fluids in a subaerial hot spring setting [4]. Moreover, the detection of minerals, specifically kaolinite and illite minerals, suggest that acid-sulfate conditions were likely present within this caldera-like environment, at the very minimum, during the final stages as this high-sulfidation volcanic system evolved [4]. Like moderately acidic springs in YNP, such as SJ3 and MV2, the acidity and sulfate in springs inferred to have existed in this ancient volcanic caldera in the Dresser Formation were likely generated by condensation of volcanic gases containing SO_2_ and H_2_S with groundwater, which can produce acidic, sulfur-rich conditions [62]. When combined with evidence suggesting that volcanic activity was more prevalent during the Archean eon (4.0–2.5 Ga) relative to today [11], these data lead to the conclusion that moderately acidic sulfur-rich springs capable of supporting ancestors or analogs of YNP-TEG likely existed on early Earth and were potentially prevalent.

The phylogenetic evidence described above, which is broadly consistent with previous analyses [49, 50], suggests that cultivated thermophiles or inferred thermophiles (*Moorella* spp.; the YNP-TEG; Crenarchaeota genera ubiquitous among hot springs including *Vulcanisaeta, Pyrobaculum, Thermoproteus*, and *Caldivirga*; the c.d. “Hydrothermarchaeota”; and c.d. “Aigarchaeota”) from hydrothermal settings harbor the earliest diverging DsrAB homologs currently known, and are thus reflective of the earliest SRO (Fig. 3b). While two other enzyme complexes, Asr and Mcc, can also reduce SO_3_^2−^ in dissimilatory metabolisms [65, 66], both exhibit patchy taxonomic distributions and are generally found in derived lineages [50], observations that together suggest them to be recently evolved. The only significant exceptions to inferred thermophily among the early-branching DsrAB homologs are from MAGs representing the c.d. “Rokubacteria” from subsurface environments and a Verrucomicrobia MAG recovered from peat soils (Fig. 3b), both of which are suggested to have acquired their putative sulfur oxidizing or SO_3_^2−^ reducing/sulfur oxidizing functions, respectively, from one or multiple horizontal transfers of DsrAB and other companion proteins [50]. Among the inferred metabolic coupling of early-diverging DsrAB: *Moorella* spp. do not reduce either SO_4_^2−^ or SO_3_^2−^ (but generally reduce S_2_O_3_^−^) rendering the function of both of their DsrAB copies unclear. In contrast, the YNP-TEG are only potentially capable of reducing SO_3_^2−^ while the Crenarchaeota SRO comprise taxa reported to reduce SO_3_^2−^ and SO_4_^2−^. The Verrucomicrobia MAG SbV1 [67] lacks Sat and AprA (confirmed with HMM searches) rendering SO_4_^2−^ reduction unlikely while only one of the two “Hydrothermarchaeota” MAGs with DsrAB (JdFR-18) contains Sat and AprAB-like proteins (JdFR-17 does not). However, the Sat and AprAB-like proteins in JdFR-18 are not genomically co-localized with either of two copies of DsrAB but are co-localized with a homolog of F_420_-linked sulfite reductases of methanogens, and the AprA-like protein lacks the characteristic residues strictly conserved among AprA homologs (Supplementary Fig. S10) This rendors  the function of both DsrAB copies to be unknown and suggests they are not likely be coupled to SO_4_^2−^ reduction. Likewise, the c.d. “Aigarchaeota” MAG that contains DsrAB does not contain AprAB or Sat. Finally, the c.d. “Rokubacteria” MAGs likely acquired sulfur oxidation potential through horizontal transfer of various Dsr proteins from various sources [50], including DsrAB potentially transferred from organisms harboring deeply-rooted DsrAB homologs like the Crenarchaea [50]. Thus, the potential for the earliest diverging DsrAB homologs to be coupled to SO_4_^2−^ reduction appears to be highly limited. A more parsimonious explanation of the above data is that the early-diverging DsrAB homologs are generally linked to SO_3_^2−^ reduction rather than SO_4_^2−^ reduction, as exemplified by the YNP-TEG MAGs.

Although SO_4_^2−^ was likely to be generally less abundant in Archean environments when SRO are thought to have originated when compared with contemporary conditions [8, 9], the possibility that SO_4_^2−^ utilizing SRO emerged in a thermal environment that hosted elevated SO_4_^2−^ is an equally plausible scenario to explain the isotopically depleted sulfides in the Dresser Formation barites. At temperatures greater than ~150 °C, volcanic SO_2_ can disproportionate to form S^0^/H_2_S and SO_4_^2−^ [15] allowing for localized enrichment of SO_4_^2−^ at concentrations capable of supporting SRO. Alternatively, SO_4_^2−^ derived from photochemical oxidation of atmospheric SO_2_ and subsequent rain-out could have also contributed SO_4_^2−^ to nonthermal surface waters [12]. Further, localized evaporation of these waters could generate still higher SO_4_^2−^ concentrations [14]. Intriguingly, it was recently shown using purified DsrAB that the primary enzymatic influence on sulfur isotope fractionation during SO_4_^2−^ reduction is at the level of reduction of SO_3_^2−^ to sulfide (via DsrAB) [68], although a fractionation effect by enzymatic SO_4_^2–^ activation and reduction (i.e., by APS reductase; AprAB) may also occur [69]. Thus, it is unclear whether SO_3_^2−^ or SO_4_^2−^ reducing organisms (or both) were responsible for the measured sulfur isotope fractionations between sulfides produced from such activities and barites in the ~3.5 Ga Dresser Formation hydrothermal deposits. An alternative explanation, and one that is consistent with the discovery of deeply diverging SO_3_^2−^ reducing (but not SO_4_^2−^ reducing) thermophilic populations described here and elsewhere, is that SO_3_^2−^ reducers were present in the Archean in volcanically-influenced environments either as predecessors or as contemporaries of SO_4_^2−^ reducers. If true, then the ultimate global dominance of SO_4_^2−^ reducers would have occurred with increasing input of SO_4_^2−^ into oceans via oxidative weathering of continental sulfide minerals. Such an explanation is consistent with previous suppositions regarding the primitive nature of SO_3_^2−^ reducers and the later dominance by SO_4_^2−^ reducers based on several lines of inference [21–23]. The buildup of atmospheric O_2_ would have allowed for the global dominance of SO_4_^2−^ reducers and also would have had the opposite effect on SO_3_^2−^ and the organisms that solely use SO_3_^2−^ as an oxidant since it is unstable in the presence of O_2_. One notable exception is anoxic or suboxic, moderately acidic and moderate thermal environments that are infused by SO_2_, such as volcanically-influenced hydrothermal environments that likely have persisted in a relatively similar state since early in Earth history.

In support of this hypothesis, SO_3_^2−^ reduction represents a simpler metabolic pathway, involves fewer enzymes, and, following the ideas of the evolution of metabolic pathways proposed by Granick [70], may therefore have originated prior to the more complex SO_4_^2−^ reduction pathway. In addition to the above evidence for the evolutionary primacy of SO_3_^2−^ reducers, the majority of the free energy yield from SO_4_^2−^ reduction arises from SO_3_^2−^ reduction to sulfide, while the first SO_4_^2−^ activation step with ATP is endergonic, and the free energy yield from APS reduction to SO_3_^2−^ is minimal [24]. The retention of the ability of many SO_4_^2−^ reducers to grow on SO_3_^2−^ combined with the considerably higher growth yields and efficiencies achieved by widely-studied SO_4_^2−^ reducers including *Desulfotomaculum orientis* [71] and *Desulfovibrio vulgaris* [72] when grown on SO_3_^2−^ relative to SO_4_^2−^, may provide additional evidence for the primitive nature of the SO_3_^2−^ reduction pathway. Consequently, the YNP-TEG archaeal group may represent descendants or analogs of primitive SO_3_^2−^ reducing organisms and important models to understand the early evolution and emergence of SO_3_^2−^ and/or SO_4_^2−^ reduction pathways. While preliminary data indicate that MV2 sediment populations can reduce SO_3_^2−^ (but not SO_4_^2−^), further efforts are needed to identify suitable conditions to maintain and propagate these cells for use in physiological, biochemical, and isotopic characterization.

## Supplementary information

Supplementary Information

Supplementary Dataset 2

Supplementary Dataset 1

Supplementary Dataset 3

Supplementary Dataset 4

## Data Availability

The MV2 and SJ3 assembled metagenomes are available in the Integrated Microbial Genomes (IMG) database under accessions 33000029569 and 3300029625, respectively. The scaffolds and the annotated gene locus tags for the MV2-Eury bin are provided in Supplementary Dataset 1. MV2-Eury MAGs from 2014 to 2018, in addition to the SJ3-Eury MAG are also available under the NCBI Bioproject accession PRJNA593284 (Biosamples SAMN13474541, SAMN13474543, and SAMN13474542, respectively).
