# Supplementary material for: Phylogenomic analysis of novel Diaforarchaea is consistent with sulfite but not sulfate reduction in volcanic environments on early Earth
Source: ISME J. 2020 Feb 17;14(5):1316–31. doi: 10.1038/s41396-020-0611-9 (PMC7174415; doi:10.1038/s41396-020-0611-9)
Supplement: Supplementary file 1 — Supplementary Information [file 41396_2020_611_MOESM1_ESM.docx]

**SUPPLEMENTARY MATERIALS**

**SUPPLEMENTARY TEXT**

**Supplementary Methods: Phylogenetic analyses**

Appropriate amino acid substitution models were chosen for each phylogenetic analysis based on Bayesian Information Criterion (BIC) scores, as implemented in the program ModelFinder (1). In each case, the LG protein substitution model provided the best-scoring phylogenetic model. Concatenated alignments were subjected to phylogenetic reconstruction with RAxML (2) and IQ-TREE (3) specifying the LG protein substitution model with a Gamma-shaped rate distribution, and evaluation of node support with maximum-likelihood (ML) bootstraps, as previously described (4). Functional genes of interest (DsrAB, AprA) were also subjected to phylogenetic reconstruction as described above using the same protein substitution models. Recently published and comprehensive DsrAB databases (5, 6) were used as reference datasets. Homolog sequences were screened from the DsrA and DsrB databases that contained less residues than the mean for either datasets (293 and 236, respectively), where the maximum number of residues in either alignment was 469 and 409, respectively, and the median values were 340 and 242, respectively. DsrA sequences were further screened based on the presence of conserved Cx_5_CX_n_CX_3_C siroheme binding motifs and CX_2_CX_2_C [Fe_4_S_4_] cluster binding motifs. Moreover, DsrA and DsrB homologs were only used that were paired with their respective subunits. Duplicate DsrAB copies within individual reference genomes were differentiated based on their locations within genomes (i.e., immediately adjacent *dsrA* and *dsrB* genes). To identify the potential root of the DsrAB homologs, paralogous rooting was conducted using an equivalent number of representative DsrA and DsrB homologs. To evaluate the potential for topological artefacts resulting from ML algorithm and substitution model choice, additional paralogous phylogenetic analyses were conducted for the DsrAB alignment using the IQ-TREE ML algorithm (3). In addition, a posterior mean site frequency (PMSF) mixture model was used to approximate site-specific amino acid profiles in order to mitigate potential long branch attraction artefacts (7).

**Supplementary Results: Metagenomic Characterization of MV2 and SJ3 Sediment Communities**

Illumina MiSeq shotgun sequencing of the sediment community from MV2 yielded a 27 Mbp assembly, and binning of contigs resulted in four moderate to high quality MAGs with an estimated >50% genome completeness (and one with 45% estimated completeness; Supplementary Table S2). The MV2 sediment community exhibited low diversity and was dominated by the MV2-Eury MAG and Archaea closely related to characterized S^0^ reducers (*Caldisphaera, Thermoproteus,* and *Acidilobus*), with minor populations of Bacteria closely related to characterized H_2_S/S^0^ oxidizers (*Hydrogenobaculum*) (Supplementary Table S2), consistent with previous 16S rRNA gene sequence community profiling of MV2 (8, 9). The details of metagenomic sequencing, assembly, and binning of SJ3 spring communities are described elsewhere (4). Briefly, 278 Mbp of assembled contigs were generated from SJ3 sediments and 82 moderate to high quality MAGs were recovered via tetranucleotide frequency- and coverage-based genomic binning. The SJ3 community was dominated by MAGs closely affiliated with the Bacteria *Sulfurihydrogenibium* (Aquificales) and *Caldisericum* spp. (Caldisericales; ~15% estimated relative abundance each), in addition to a wide diversity of lower abundance archaeal and bacterial MAGs exhibiting considerable metabolic diversity (4).

**Supplementary Results: Core Metabolism of the YNP-TEG Group**

The YNP-TEG MAGs encoded a co-localized *mvhAGD* [NiFe]-hydrogenase operon (Group 3c electron bifurcating type) but no other hydrogenases were present in the assemblies (Fig. 2). HdrA, the flavin-binding, ferredoxin (Fd)-reducing, and presumed electron bifurcating subunit of Mvh/HdrABC complexes was co-localized with the *mvh* operons. However, homologs of HdrB (which function in the reduction of disulfides) and HdrC homologs were not detected in the MAGs. Thus, it is likely that the YNP-TEG MvhADG/HdrA oxidize H_2_ to reduce Fd. However, it is unclear how electrons are bifurcated to another oxidant, which must be accomplished in order to drive the thermodynamically unfavorable reduction of Fd with H_2_. Regardless, *mvhAGD-hdrA* (without *hdrBC*) are prevalent among SRO genomes (10), and likely function in coupling the reduction of an unidentified oxidant with H_2_ through electron bifurcation.

In addition, the YNP-TEG MAGs encoded several pathways to produce Fd_red_ from oxidation of organic carbon substrates (e.g., through Aor, aldehyde ferredoxin oxidoreductase; Ior, indolepyruvate ferredoxin oxidoreductase; Vor, 2-ketoisovalerate ferredoxin reductase; Kgor, 2-ketoglutarate ferredoxin oxidoreductase, and Por, pyruvate ferredoxin oxidoreductase). The Fd_red_ from these various pathways are then likely oxidized thereby driving proton translocation via an archaeal-type Fd-dependent Nuo_A_ complex. The catalytic subunit homolog (NuoD) of the Nuo_A_ complex within the YNP-TEG MAGs lacked the diagnostic cysteines that coordinate the [NiFe] hydrogenase in FpoD/MbxL, suggesting that it belongs to the Nuo_A_ group (i.e., lacking NuoEFG as in bacterial-type Nuo_B_) (11).

PCGs necessary for canonical CO_2_ fixation pathways (Acl, Ccs, RuBisCo, abfD, and malonyl-CoA reductase) were not present in the MAGs. However, homologs comprising a hypothesized chimeric CO_2_ fixation pathway that incorporates components of the Wood-Ljungdahl CO_2_ fixation pathway and the serine/glycine cycle, were detected in the YNP-TEG MAGs. The chimeric pathways have been hypothesized to explain mixotrophic growth in Ferroplasmatales Archaea (12) and Deltaproteobacteria (13). Nevertheless, this pathway has only been proposed from genomic data to explain mixotrophic growth in the absence of canonical CO_2_ fixation pathways and thus requires further biochemical and physiological characterization. Consequently, it is unclear if the YNP-TEG groups are autotrophic, heterotrophic, or mixotrophic.

BLASTp searches and KEGG-based annotations revealed that the MAGs lacked evidence for known pathways to oxidize sulfide or sulfur including the absence of flavocytochrome C (FccAB; WP_012970308 and 012970307 from *Allochromatium* *vinosum* used for BLAST search) subunits used for sulfide oxidation, sulfur oxidoreductase (Sox) subunits used in sulfur oxidation within many taxa, sulfide-quinone reductase (Sqr; WP_012536761 from *Acidithiobacillus ferrooxidans*), sulfite dehydrogenase used in SO_3_^2-^ oxidation (SoeABC), and sulfur oxygenase used in sulfur oxidation (Sor). A putative sqr was identified by KEGG mapping (Ga0311280_10002626) that upon further scrutiny was revealed to more broadly be a member of the pyridine nucleotide-disulphide oxidoreductase protein family, with closest homologs in archaeal organisms not known to oxidize sulfide.

**Supplementary Results: The presence of DsrAB in *Moorella* spp.**

As in previous analyses (5, 6), a second genomic copy of DsrAB from the bacterial genus *Moorella* (Firmicutes) formed an outgroup to both DsrA and DsrB subtrees (Fig. 3). *Moorella*, like the crenarchaeal and YNP-TEG SRO are thermophilic and many have also been isolated from thermal springs, including those in YNP (14). Although *Moorella* harbor two genomic copies of DsrAB, no currently described *Moorella* isolates are capable of either SO_3_^2-^ or SO_4_^2-^ reduction when supplied as electron acceptors, but nearly all are capable of using S_2_O_3_^-^ for energy conservation (14, 15). Thus, while the function of the second DsrAB copies in *Moorella* genomes is not known, it is likely that they are functionally distinct from the other DsrAB copies in *Moorella* genomes, which share high homology to reductive DsrAB from other Firmicutes. In support of this interpretation, the genes that encode the Firmicutes-like DsrAB from *Moorella* are co-localized with other genes necessary for dissimilatory SO_4_^2-^ reduction (e.g., *dsrD* and *dsrMKJOP*), while the deep-branching second copies are not co-localized with other genes involved in reduction of SO_4_^2-^ (e.g., IMG gene IDS: 2705923090 and 2705908875). Thus, the deep branching DsrAB homologs from *Moorella* spp. may have a function that is distinct from dissimilatory SO_3_^2-^/SO_4_^2-^ reduction such as SO_3_^2-^ detoxification. Further physiological and/or biochemical characterizations will be necessary to confirm the functions of these homologs.

**Supplementary Results: Apr-like Homologs in the YNP-TEG Assemblies**

Adenylylsulfate reductases (Apr) belong to a large family of oxidoreductases that includes succinate dehydrogenases, fumarate reductases, and L-aspartate oxidases (16). AprA-like proteins annotated as L-aspartate oxidases were identified in the MV2-Eury and SJ3-Eury assemblies via BLAST and HMM-based searches, but lacked significant homology to those from canonical SRO and S^0^ oxidizers in addition to the characteristic conserved residues associated with bona fide AprA (16). Nevertheless, the proteins were subjected to phylogenetic analysis along with AprA from canonical SRO and the closest homologs/paralogs present in public genomic databases. In addition, the gene neighborhood contexts of the AprA-like homologs/paralogs were characterized to evaluate their putative functions. The AprA-like genes present in the YNP-TEG assemblies were present in either non-SO_4_^2-^ reducer genomes or within duplicate genomic copies in SO_4_^2-^ reducer genomes, and were primarily associated with ion transporters and regulatory proteins (Supplementary Figure S10). To our knowledge, this protein group has not been functionally characterized, but it is unlikely that they operate in SO_4_^2-^ reduction due to the lack of characteristic residues of AprA, their genomic context, and distinction from canonical *aprAB* that genomically co-localize with other SO_4_^2-^ reduction genes (*qmoABC, sat*) (Supplementary Figure S10). Moreover, the AprA-like proteins of the YNP-TEG groups lacked the characteristic glycine, arginine, tryptophan, and tyrosine residues that are strictly conserved among AprA (Supplementary Figure S10; (16)), consistent with the annotation of the AprA-like protein from MV2-Eury as an L-aspartate oxidase.

**Supplementary Results: Absence of Previously Suggested Marker Genes for SO_4_^2-^ Reduction and S^0^ Oxidation**

The MV2-Eury and SJ3-Eury MAGs were screened for marker genes that demarcate SRO from organisms that oxidize S^0^. *dsrD* is typically absent in the genomes of DsrAB-encoding organisms that oxidize S^0^ but is nearly universally present in the genomes of SRO (6). *dsrD* was absent in the YNP-TEG genomes, which is consistent with its absence in other Crenarchaeota that have been shown to reduce SO_4_^2-^/SO_3_^2-^, and its absence in the deep branching DsrAB lineage represented by ‘Rokubacteria’ MAGs, ‘Hydrothermarchaeota’ MAGs, and a single Verrucomicrobia MAG (6). Similarly, homologs of DsrEFH, which function in concert with DsrAB during S^0^ oxidation (17) and which have been suggested as marker genes for DsrAB-mediated S^0^ oxidation (6), were not observed in the YNP-TEG assemblies. Although two potential DsrE/F orthologs were identified in the YNP-TEG assemblies via HMM searches, one lacked the conserved Cys78 residue considered essential for DsrE activity (18), while the other affiliated with the peroxiredoxin protein family that is related to the DsrE protein family (18) and widespread in Archaea.

**Supplementary Figures**

**
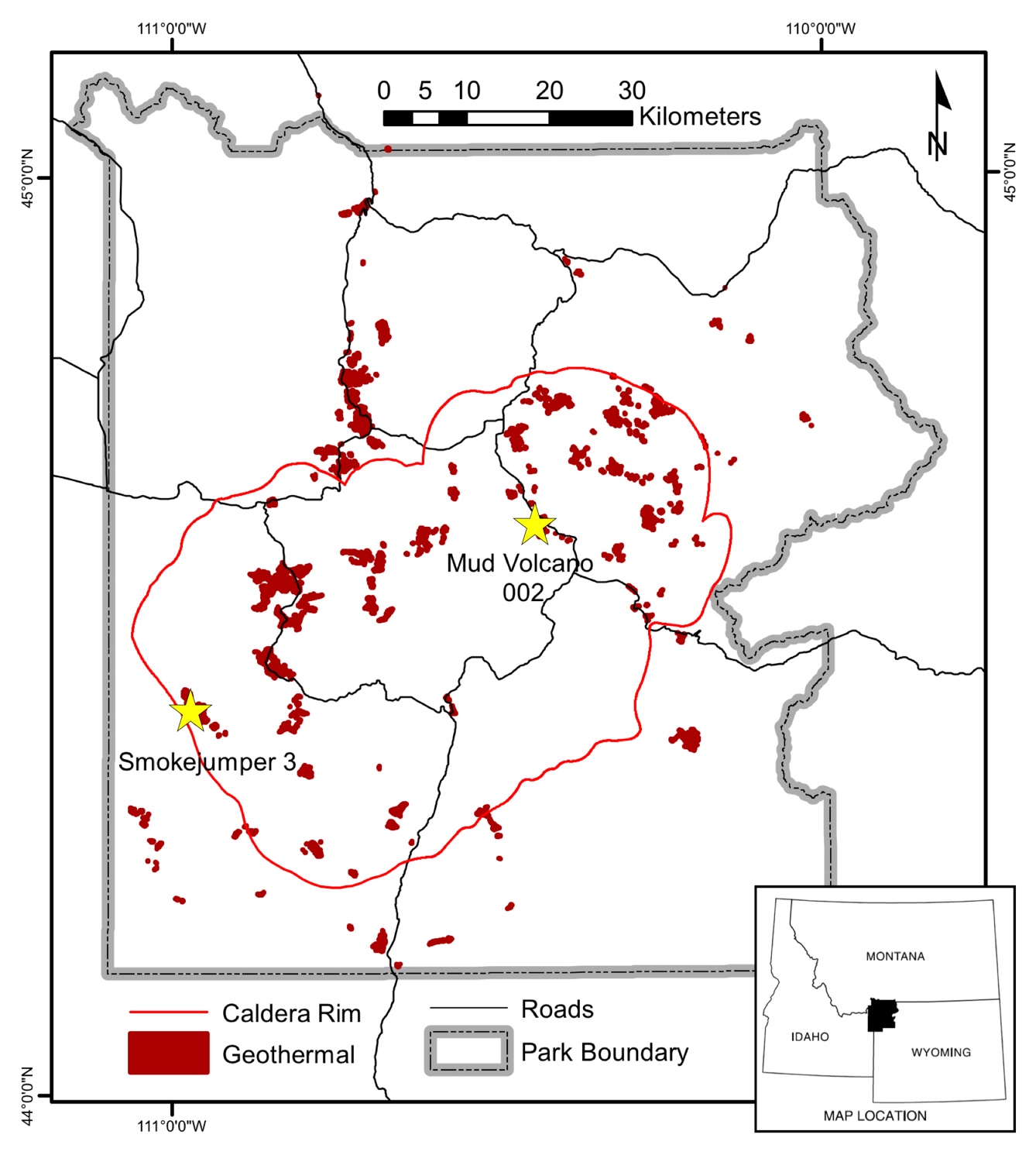
**

**Supplementary Figure S1. Map of Yellowstone National Park (YNP) showing the location of Mud Volcano 002 (MV2) and Smokejumper 3 (SJ3) hot springs and photos of MV2 and SJ3 springs.** The caldera rim and geothermal area layers are adapted from the United States Geological Survey (USGS) Geographic Information Systems (GIS) database and (19). Park boundary and road reference layers are taken from the USGS GIS database. Top photo shows MV2 spring in July 2018. Bottom photo shows SJ3 spring in July 2014. White stars indicate sampling locations within each spring.

**Supplementary Figure S2. Alignment of MV2/SJ3-Eury-specific *dsrA* primers and selected *dsrA* homologs.** Alignments were conducted using Clustal, as described in the materials and methods. The number of mismatches for each primer and each *dsrA* homolog, along with the theoretical product sizes are shown to the right of the alignments.

**Supplementary Figure S3. Sulfate and chloride values of MV2 and SJ3 in the context of those for other YNP springs.** Sulfate and chloride data from hot springs in YNP as reported elsewhere (20) are plotted as grey circles (n=488). SJ3 is plotted in red while MV2 is plotted in green. SJ3 data are from Lindsay *et al.* 2019 (20) and those for MV2 are from Colman 2015 (8). Overlaid on the plot are source water designations from Nordstrom *et al.* 2009 (21). HO=hydrothermal only waters are theorized to be sourced from a deep hydrothermal reservoir, MO=dilute meteoric or precipitation derived waters, and MG=acid-sulfate waters represent meteoric waters influenced by vapor-phase gas inputs. Springs that are right-shifted from the HO group represent deep waters that are interpreted to have also undergone subsurface boiling and evaporation resulting in a higher concentration of chloride (hydrothermal fluids with subsurface boiling in (21)).

**
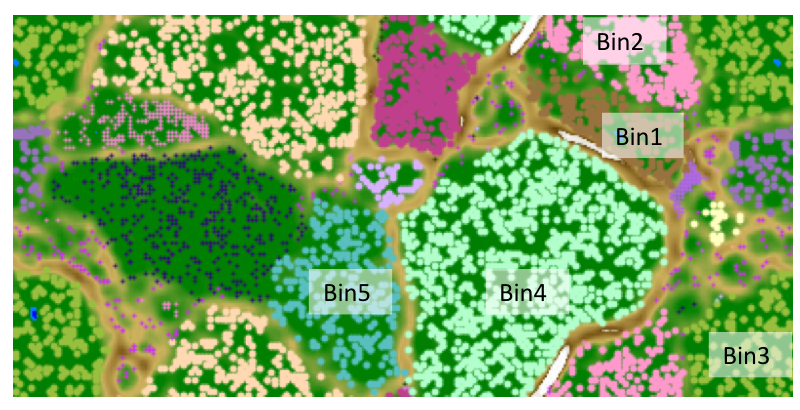
**

**Supplementary Figure S4. Emergent self organizing map (ESOM) for the MV2 sediment community from 2014.** Each point represents a contig >2,500 bp from either a reference genome or an MV2 community MAG. The five bins described in Supplementary Table S2 are labeled in the figure. Groups were delineated based on ESOM analysis of tetranucleotide frequencies, as indicated by distance-based borders in brown. Small dark purple points are contigs not incorporated into groups and non-labeled groups are reference genomes used to better delineate MV2 community bins. All MV2 bins exhibited < 2.0% estimated redundancy (i.e., ‘contamination’), with most exhibiting no redundancy at all, as indicated by quality analysis in CheckM (Supplementary Table S2).

**
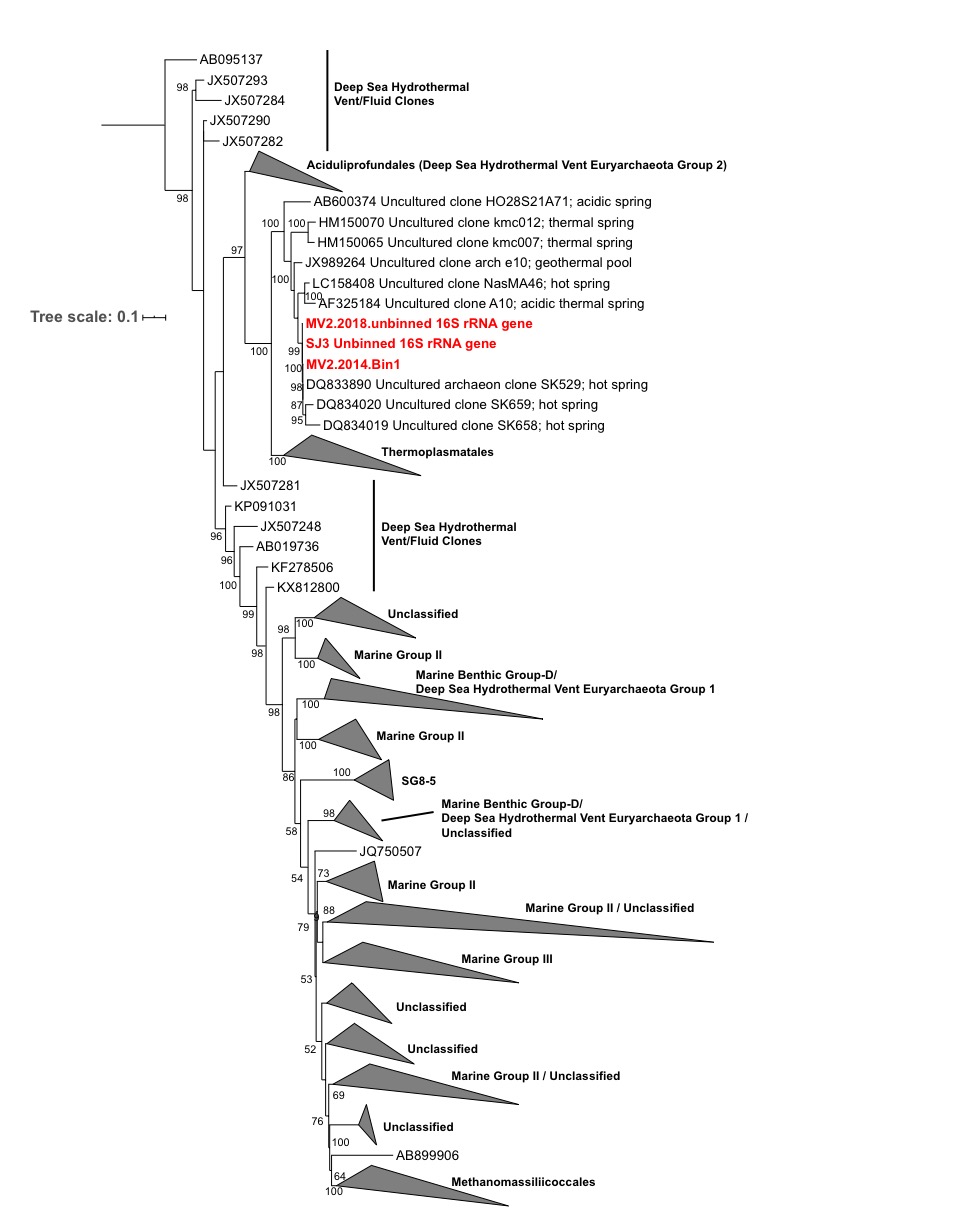
**

**Supplementary Figure S5. Maximum likelihood phylogenetic reconstruction of 16S rRNA genes representing the MV2/SJ3-Eury MAGs and other Diaforarchaea**. The National Center for Biotechnological Information (NCBI) accession number for each reference is shown prior to the 16S rRNA gene identifiers. A 16S rRNA gene for *Candidatus* Korarchaeum cryptofilum was used as the outgroup (not shown). All reference 16S rRNA genes >900 bp classified within the ‘Thermoplasmata’ in the most recent Silva database release were used as references, in addition to all other near full-length 16S rRNA genes present within the NCBI database showing close affiliation with the YNP-TEG populations. To reduce the size of the dataset, 16S rRNA genes were first clustered into operational taxonomic units at the 97% nucleotide identity level, and representatives were chosen from each OTU to include in the analyses (n=1,533 total sequences after filtering). Phylogenetic analysis of de-replicated OTUs for those closely affiliated with the MV2/SJ3-Eury 16S rRNA genes (e.g., those within the YNP-TEG group) are shown in Supplementary Figure S6. Environments of origin are given for those 16S rRNA gene entries that monophyletically affiliated with the YNP-TEG group. Other taxonomic designations are given based on the Silva database taxonomic classifications. 16S rRNA genes associated with the MV2- and SJ3-Eury MAGs are highlighted in red. Branch length is relative to the scale provided at the left indicating the expected number of substitutions per site. Bootstrap values >50 are shown at nodes (out of 1,000 replicates). The clade-level triangles indicate the phylogenetic diversity within each group via side lengths that are proportional to the distances between the clade’s closest and furthest taxa.

**Supplementary Figure S6. Maximum likelihood phylogenetic reconstruction of 16S rRNA genes representing the YNP-TEG MAGs and other closely-related phylotypes**. The National Center for Biotechnological Information (NCBI) accession number for each reference is shown prior to the 16S rRNA gene identifiers. A 16S rRNA gene for *Thermoplasma volcanium* was used as the outgroup (not shown). All reference 16S rRNA genes >900 bp classified within the ‘Thermoplasmata’ in the most recent Silva database release were used as references, in addition to all other near full-length 16S rRNA genes present within the NCBI database showing close affiliation with the YNP-TEG populations. The 16S rRNA gene entries shown here are a subset of the entire Diaforarchaea dataset shown in Supplementary Figure S5. Environments of origin are given for all entries based on metadata provided in the genbank accession metadata. 16S rRNA genes associated with the MV2- and SJ3-Eury MAGs are highlighted in red. Branch length is relative to the scale provided at the top of the tree indicating the expected number of substitutions per site. Bootstrap values >50 are shown at nodes (out of 1,000 replicates).

**
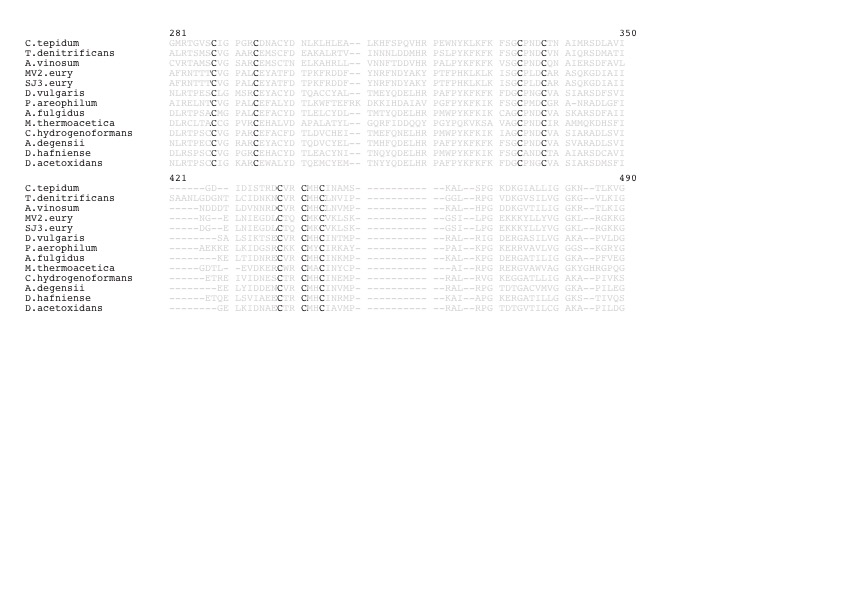
**

**
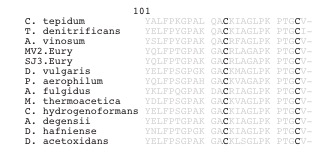
**

**Supplementary Figure S7. Sequence alignment of the YNP-TEG DsrA homologs (top) and DsrC (bottom) homologs from other model SRO or sulfur oxidizing bacteria.** Conserved cysteine motifs involved in siroheme binding (CX_5_CX_n_CX_3_C) are shown in the DsrA upper alignment block in non-shaded regions, while those of the character­­istic [Fe_4_S_4_] cluster binding motif (CX_2_CX_2_C) are shown in the lower alignment block. Conserved cysteine residues in the C-terminal arm of DsrC are shown in non-shaded regions in the bottom panel.

**
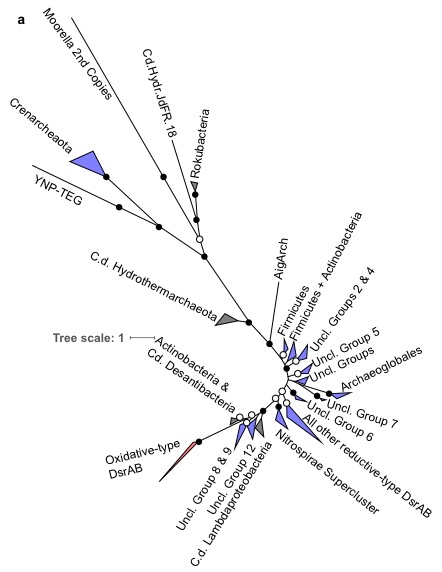
**

**Supplementary Figure S8. Unrooted Maximum Likelihood phylogenetic reconstruction of YNP-TEG DsrAB subunit homologs.** The phylogenetic reconstruction is the same as shown in Fig. 3A, but without midpoint rooting. Unrooted phylogenetic reconstruction of a concatenated DsrAB alignment block (alignment length = 1,230 positions, n=1,218 homologs). Branches are colored according to major taxonomic/functional groups (following the classification scheme of (5)) wherein light blue denotes inferred reductive-type DsrAB, red denotes inferred oxidative-type DsrAB, and dark grey denotes uncertainty in DsrAB directionality in recently discovered uncultured lineages. Inferred directionality follows annotations from previous publications (5, 6)*.* Bootstrap values > 90 for clade-level bifurcations are indicated by black circles while those between 50 and 90 are denoted by white circles. Values are not depicted for nodes with bootstrap support < 50. Branch length is relative to the scale provided at the bottom indicating the expected number of substitutions per site. The clade-level triangles indicate the phylogenetic diversity within each group via side lengths that are proportional to the distances between the clade’s closest and furthest taxa.

**
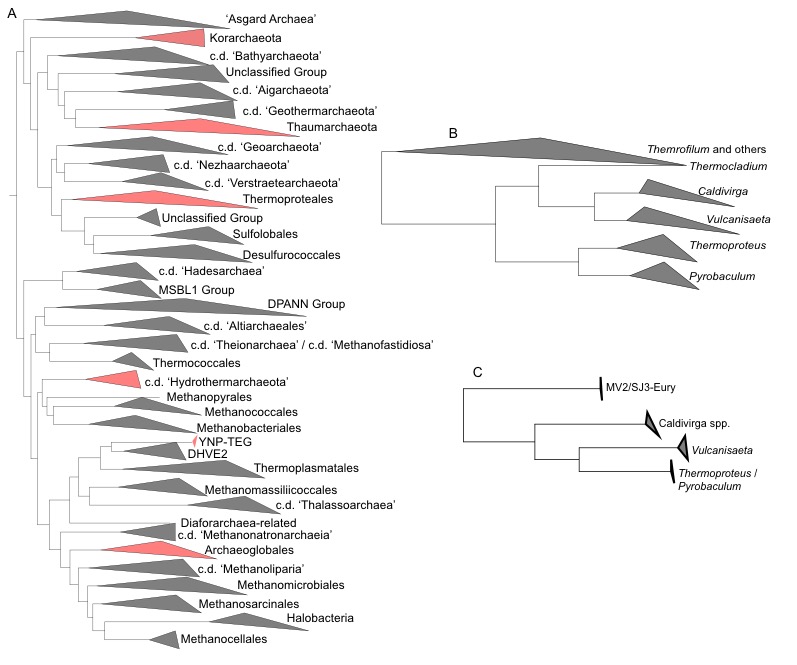
**

**Supplementary Figure S9. Phylogenetic context of the YNP-TEG group within Archaea. A)** Unrooted phylogenomic tree of the major divisions of Archaea. The Maximum Likelihood tree is based on a concatenation of 103 house-keeping marker genes as described in the materials and methods. Clades highlighted in red have been shown to harbor organisms with DsrAB. **B)** Tree subset shows the Thermoproteales group within Crenarchaeota. **C)** Concatenated DsrAB tree subset showing the placement of MV2/SJ3-Eury DsrAB homologs and those of Thermoproteales. Bootstraps for all nodes shown >99. The clade-level triangles indicate the phylogenetic diversity within each group via side lengths that are proportional to the distances between the clade’s closest and furthest taxa.

**
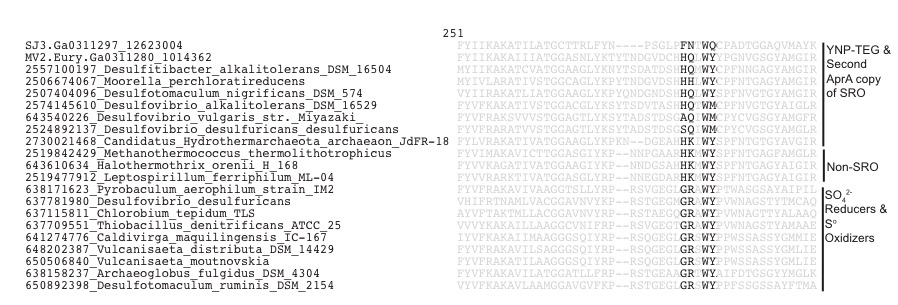
**

**Supplementary Figure S10. Maximum likelihood phylogenetic reconstruction of the MV2-Eury adenosine 5′-phosphosulfate reductase (AprA)-like paralogs with closest homologs and AprA paralogs from characterized SRO and sulfur oxidizers.** Each AprA homolog is identified by its Integrated Microbial Genome gene accession number and the organism it is derived from. In addition, all identifiable AprA homologs from the MV2 metagenome are preceded by the metagenome identifier) (Ga0311280), and the same are shown from homologs from the SJ3 metagenome (Ga0311297). Bootstrap values are given at the nodes, and the scale bar at the top left depicts the expected number of substitutions per site. The presence of protein homologs necessary for SO_4_^2-^/SO_3_^2-^ reduction is shown in the first three columns to the right for 1) the presence of DsrA and DsrB, 2) Sat, and 3) additional AprA-like homologs in the corresponding genomes. Black circles indicate positive detection, while white circles indicate the lack of detection. The capacity for SO_3_^2-^ , S_2_O_3_^-^, or SO_4_^2-^ reduction is also shown, based on characterized strains corresponding to the genomes indicated. Question marks indicate that the substrate was not tested during strain characterization. Lastly, representative gene neighborhood representations are shown for AprA homologs and their corresponding duplicate homologs, if present within a genome. Blue: genomic region consists of 1) an *aprB*-like homolog, 2) an anion transporter (typically annotated as a sulfate/sodium symporter) or tricarboxylate transporter and 3) a regulatory protein annotated as a cAMP-binding domain of CRP (COG0664). Red indicates the presence of 1) an *aprB*-like homolog and 2) *sat* and/or *qmoABC*. Green indicates a gene neighborhood without any of the above associated homologs. Additional information for each homolog is provided in Supplementary Dataset 4. AprA and AprA-like gene alignment is shown below the figure for select AprA homologs from the ML tree, with the glycine, arginine, tryptophan, and tyrosine residues conserved in AprA (16) shown within the unshaded regions.

**
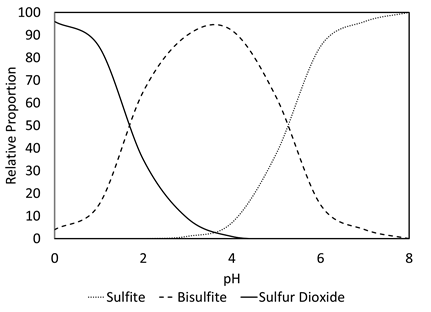
**

**Supplementary Figure S11. Relative proportions of ionization products of sulfurous acid in waters with specified pH.** Data are adapted from (22). Sulfite: SO_3_^2-^; bisulfite: HSO_3_^-^; sulfur dioxide: SO_2_.

**Supplementary Tables**

**Supplementary Table S1. Select field and geochemical measurements for SJ3 and MV2 springs from the present study and previously published studies.**

| **Spring** | **Date** | **Temperature** | **pH** | **Conductivity (mS)** | **SO_4_^2-^ (mg/L)** | **Cl^-^ (mg/L)** |
| --- | --- | --- | --- | --- | --- | --- |
| SJ3 | 7/22/14 | 61.9 | 5.4 | 0.42 | 70.32 | 1.05 |
| MV2 | 10/22/18 | 65.9 | 3.7 | 3.95 | 346.7 | 380 |
| MV2 | 7/27/18 | 67.2 | 3.5 | 4.06 | nd | nd |
| MV2 | 7/19/18 | 68.7 | 3.7 | 4.12 | nd | nd |
| MV2 | 6/11/18 | 58.7 | 3.7 | 3.67 | nd | nd |
| MV2 | 7/14/17 | 67.7 | 3.3 | 4.28 | nd | nd |
| MV2 | 11/4/16 | 66.6 | 3.0 | 3.80 | 370 | nd |
| MV2 | 7/24/16 | 67.4 | 3.2 | 2.40 | nd | nd |
| MV2^a^ | 7/14/14 | 62.0 | 3.8 | 4.26 | nd | nd |
| MV2^b^ | 7/29/10 | 61.3 | 4.8 | 2.26 | 306.81^b^ | 373.31^b^ |
| MV2^c^ | 7/20/04 | 65.0 | 4.0 | nd | 348.7^c^ | nd^c^ |
| MV2^c^ | 7/9/04 | 65.0 | 3.1 | nd | 397.69^c^ | nd^c^ |

^a^Data from Colman *et al.* (2016) (9)

^b^Data from Colman (2015) (8)

^c^Data from Romano *et al.* (2013) (23)

**Supplementary Table S2. Metagenome assembled genome (MAG) statistics from the MV2 spring community metagenome.**

|  | **Bin.1** | **Bin.2** | **Bin.3** | **Bin.4** | **Bin.5** |
| --- | --- | --- | --- | --- | --- |
| Est. Completeness | 97.58 | 78.48 | 97.02 | 77.27 | 45.57 |
| Est. Contamination | 0.81 | 0.00 | 2.13 | 0.00 | 0.00 |
| Size (Mbp) | 1.30 | 1.33 | 1.62 | 1.28 | 0.91 |
| No. of Contigs | 34 | 73 | 110 | 202 | 108 |
| Largest Contig (Kbp) | 354.76 | 152.91 | 115.75 | 24.96 | 238.17 |
| Predicted Protein Coding Genes | 1,384 | 1,362 | 1,776 | 1,628 | 1,096 |
| Estimated Relative Abundance | 6.4% | 40.5% | 4.9% | 3.3% | 33.7% |
| Taxonomic Affiliation (Order) | Diaforarchaea | *Caldisphaera* (Desulfuro-ccoccales) | *Hydrogenobaculum* (Aquificales) | *Thermoproteus* (Thermo-proteales) | *Acidilobus* (Desulfuro-coccales) |
| Nearest Cultivated Taxon by 16S rRNA gene | n/a | *Caldisphaera draconis* 18U65 | *Hydrogenobaculum* sp. Y04AAS1 | *Thermoproteus tenax* | *Acidilobus sulfurireducens*18D70 |
|  |  |  |  |  |  |
| 16S rRNA Gene ID / Binned / Unbinned | n/a | 99% / binned | 99% / unbinned | 99% / unbinned | 99% / unbinned |
| Inferred Metabolism | n/a | S^0^ reducer | S^0^ oxidizer | S^0^ reducer | S^0^ reducer |

**Supplementary Table S3. Assembly statistics for the Diaforarchaea-related MV2 and SJ3 MAGs.**

|  | **MV2.2014.Bin1** | **SJ3.Bin7** | **MV2.2018.Bin2** |
| --- | --- | --- | --- |
| Estimated completeness | 98.1% | 98.1% | 98.1% |
| Estimated contamination | 0.9% | 0.9% | 0.9% |
| Assembled size (Mbp) | 1.30 | 1.19 | 1.40 |
| Longest contig (Kbp) | 354.8 | 59.4 | 91.8 |
| Contig number | 34 | 74 | 94 |
| N50 (Kbp) | 157.1 | 25.5 | 28.7 |
| Protein coding genes | 1,381 | 1,254 | 1,536 |
| G+C % | 35.7 | 36.0 | 35.9 |
| Estimated relative abundance | 6.4% | 2.8% | 8.3% |

**Supplementary Table S4. Metadata associated with 33 chemosynthetic YNP hot spring samples used to survey the presence of YNP-TEG-like organisms.**

**SUPPLEMENTARY DATASETS**

**Supplementary Dataset 1.** Protein homologs present in the MV2-Eury MAG that were used to infer the metabolic model proposed for the YNP-TEG MAGs (XLS file).

**Supplementary Dataset 2.** Newick formatted DsrAB maximum likelihood phylogeny for 1,218 DsrAB homologs.

**Supplementary Dataset 3.** AprA and Sat homologs identified in the MV2 and SJ3 metagenome assemblies (XLS file).

**Supplementary Dataset 4.** Additional information for AprA-like homologs and paralogs highlighted in Supplementary Figure 10 including gene context, the genomes they derive from, the presence of other sulfate/sulfite reduction enzymes in the genome, and the characterized metabolism of the organism (XLS file).

**Supplementary References**

1. Kalyaanamoorthy S, Minh BQ, Wong TKF, von Haeseler A, Jermiin LS. ModelFinder: fast model selection for accurate phylogenetic estimates. Nat Methods. 2017;14(6):587-9.

2. Stamatakis A. RAxML version 8: a tool for phylogenetic analysis and post-analysis of large phylogenies. Bioinformatics. 2014;30(9):1312-3.

3. Nguyen LT, Schmidt HA, von Haeseler A, Minh BQ. IQ-TREE: a fast and effective stochastic algorithm for estimating maximum-likelihood phylogenies. Mol Biol Evol. 2015;32(1):268-74.

4. Colman DR, Lindsay MR, Boyd ES. Mixing of meteoric and geothermal fluids supports hyperdiverse chemosynthetic hydrothermal communities. Nat Commun. 2019;10(681).

5. Muller AL, Kjeldsen KU, Rattei T, Pester M, Loy A. Phylogenetic and environmental diversity of DsrAB-type dissimilatory (bi) sulfite reductases. ISME J. 2015;9(5):1152-65.

6. Anantharaman K, Hausmann B, Jungbluth SP, Kantor RS, Lavy A, Warren LA, et al. Expanded diversity of microbial groups that shape the dissimilatory sulfur cycle. ISME J. 2018;12(7):1715-28.

7. Wang HC, Minh BQ, Susko E, Roger AJ. Modeling site heterogeneity with posterior mean site frequency profiles accelerates accurate phylogenomic estimation. Syst Biol. 2018;67(2):216-35.

8. Colman DR. Diversity of understudied archaeal and bacterial populations of Yellowstone National Park: from genes to genomes. Albuquerque, NM: University of New Mexico; 2015.

9. Colman DR, Feyhl-Buska J, Robinson KJ, Fecteau KM, Xu H, Shock EL, et al. Ecological differentiation in planktonic and sediment-associated chemotrophic microbial populations in Yellowstone hot springs. FEMS Microbiol Ecol. 2016;92(9).

10. Pereira IAC, Ramos AR, Grein F, Marques MC, da Silva SM, Venceslau SS. A comparative genomic analysis of energy metabolism in sulfate reducing bacteria and archaea. Front Microbiol. 2011;2.

11. Schut GJ, Zadvornyy O, Wu CH, Peters JW, Boyd ES, Adams MWW. The role of geochemistry and energetics in the evolution of modern respiratory complexes from a proton-reducing ancestor. Bba-Bioenergetics. 2016;1857(7):958-70.

12. Cardenas JP, Martinez V, Covarrubias P, Holmes DS, Quatrini R. Predicted CO/CO_2_ fixation in *Ferroplasma* spp. via a novel chimaeric pathway. Adv Mat Res. 2009;71-73:219-22.

13. Figueroa IA, Barnum TP, Somasekhar PY, Carlstrom CI, Engelbrektson AL, Coates JD. Metagenomics-guided analysis of microbial chemolithoautotrophic phosphite oxidation yields evidence of a seventh natural CO_2_ fixation pathway. Proc Natl Acad Sci U S A. 2018;115(1):E92-E101.

14. De Vos P, Garrity GM, Jones D, Krieg NR, Ludwig W., Rainey FA, et al. The Firmicutes. In: Whitman WB, Parte, A.C., editor. Bergey’s Manual of Systematic Bacteriology. 3. New York: Springer; 2009.

15. Balk M, Weijma J, Friedrich MW, Stams AJ. Methanol utilization by a novel thermophilic homoacetogenic bacterium, *Moorella* *mulderi* sp. nov., isolated from a bioreactor. Arch Microbiol. 2003;179(5):315-20.

16. Hipp WM, Pott AS, Thum-Schmitz N, Faath I, Dahl C, Truper HG. Towards the phylogeny of APS reductases and sirohaem sulfite reductases in sulfate-reducing and sulfur-oxidizing prokaryotes. Microbiology. 1997;143 ( Pt 9):2891-902.

17. Dahl C, Engels S, Pott-Sperling AS, Schulte A, Sander J, Lubbe Y, et al. Novel genes of the dsr gene cluster and evidence for close interaction of Dsr proteins during sulfur oxidation in the phototrophic sulfur bacterium *Allochromatium* *vinosum*. J Bacteriol. 2005;187(4):1392-404.

18. Dahl C, Schulte A, Stockdreher Y, Hong C, Grimm F, Sander J, et al. Structural and molecular genetic insight into a widespread sulfur oxidation pathway. J Mol Biol. 2008;384(5):1287-300.

19. Christiansen RL. The Quaternary and Pliocene Yellowstone Plateau volcanic field of Wyoming, Idaho, and Montana. U.S.G.S. Report. 2001. Report No.: 729G.

20. Lindsay MR, Colman DR, Amenabar MJ, Fristad KE, Fecteau KM, Debes RV, et al. Probing the geological source and biological fate of hydrogen in Yellowstone hot springs. Environ Microbiol. 2019;21(1):3816-3830.

21. Nordstrom DK, McCleskey RB, Ball JW. Sulfur geochemistry of hydrothermal waters in Yellowstone National Park: IV Acid-sulfate waters. Appl Geochem. 2009;24:191-207.

22. Saunders PJW, Wood CM. Sulphur dioxide in the environment: its production, dispersal and fate. In: Ferry BWea, editor. Air Pollution and Lichens. Toronto, CA: University of Toronto Press; 1973. p. 6-37.

23. Romano C, D'Imperio S, Woyke T, Mavromatis K, Lasken R, Shock EL, et al. Comparative genomic analysis of phylogenetically closely related *Hydrogenobaculum* sp. isolates from Yellowstone National Park. Appl Environ Microbiol. 2013;79(9):2932-43.
